# Distance-dependent patterns of intercity passenger mode share: A multiscale analysis of socioeconomic and spatiotemporal heterogeneity

**DOI:** 10.1371/journal.pone.0352112

**Published:** 2026-09-17

**Authors:** Yun Xiang, Tao Zeng, Weijie Yu, Longfei Ye, Wei Wang, Wenyun Tang

**Affiliations:** 1 College of City Construction, Jiangxi Normal University, Nanchang, China; 2 School of Transportation, Southeast University, Nanjing, China; 3 School of Civil and Transportation Engineering, Hebei University of Technology, Tianjin, China; 4 School of Architecture and Engineering, Jiangxi Modern Polytechnic College, Nanchang, China; 5 College of Automobile and Traffic Engineering, Nanjing Forestry University, Nanjing, China; Shanghai University, CHINA

## Abstract

The intrinsic attributes of different transportation modes render travel distance a major factor of intercity passenger mode share; yet systematic examination of how this distance effect varies across multiscale contexts remains limited. Therefore, this study investigates how travel distance regulates the shares of highway, railway, and aviation modes in intercity transportation system, and examines the underlying dynamics across multiscale socioeconomic and spatiotemporal contexts. Using nationwide Location-based Services (LBS) data collected from China, polynomial fitting models were constructed to establish the relationship between travel distance and multi-modal shares. Our results demonstrate the significant role of travel distance in shaping intercity passenger mode shares, with effects that vary substantially across different contexts. Temporally, highway mode share rises for short-to-medium trips during holiday periods such as Spring Festival Travel Rush, while aviation always dominates for long trips. Spatially, Northeast China exhibits railway dominance, coastal regions concentrate aviation flows, while Southwest China depends on highways for short-distance connectivity. From the socioeconomic perspective, urban areas with higher economic level, administrative rank, and larger population exhibit elevated aviation shares and diminished highway shares. Our empirical findings advance understanding of nonlinear distance-modal share relationships considering socioeconomic and spatiotemporal features, thereby informing multimodal transport planning and cross-regional resource optimization in intercity transportation system.

## Introduction

The ongoing development of regional transport infrastructures constitutes a core support for promoting regional coordination and facilitating intercity factor mobility. As multimodal networks, including highways, railways, and aviation, are progressively established, the resulting time-space compression strengthens connections between cities, thereby stimulating the rapid increase in intercity travel demand [1,2]. Taking China as an example, the passenger traffic of highways, railways, and aviation reached 11,011.53, 3,854.50, and 619.58 million, respectively, in 2024 (Data source: National Bureau of Statistics of China, China Statistical Yearbook 2025). Moreover, the diversification of transportation supply allows travelers to choose among multiple modes rather than being confined to a single option for intercity trips [3]. The competitive dynamics among different transport modes (e.g., highways, railways, and aviation) have become increasingly evident, driven by their diverse advantages in operating natures like speed, cost, and comfort [4]. Against this backdrop, it is essential to understand the modeling principles that shape modal shares of intercity transport modes. Such research holds significant theoretical and practical value for optimizing transportation resource allocation, improving the operational efficiency of intercity transportation, and supporting the scientific formulation of regional transportation plans.

Existing studies have widely explored the determinants of intercity modal shares and identified individual attributes as core influential factors, encompassing income, age, vehicle ownership, and even education level [5]. These attributes shape travelers’ preferences and affordability constraints, thereby exerting a pivotal impact on modal choices [6,7]. For instance, elderly travelers prioritize convenience, favoring highway or high-speed rail (HSR) [8], while vehicle owners exhibit a heightened propensity for self-driving on short-distance trips due to flexibility advantages [9]. High-income groups, with stronger willingness to pay for time-saving and high-quality services, tend to choose air or HSR [10], a trend further validated by Li et al. [6] in tourist cities where online ticketing accessibility also mediates mode selection. Altieri et al. [10] refined this understanding by categorizing travelers into choice riders (private vehicle owners), captive riders (public transport dependent), and captive-by-choice riders, noting that vehicle ownership negatively affects the modal share of public transport. Complementing this, Yang et al. [8] highlighted travelers’ age and vehicle ownership as key moderators in HSR-air competition, while Guo et al. [11] added that higher-educated groups may prefer driving for privacy concerns. Collectively, these findings underscore the multifaceted role of individual attributes in shaping intercity modal shares.

Moreover, travel characteristics, particularly distance and purpose, dominate mode selection as they directly match travel demands with the advantages of different transport modes [12]. While commuting or short-distance business trips tend to rely on high-speed rail due to their higher requirements for reliability and punctuality [13,14]. For example, mandatory trips such as business or medical travel prioritize punctuality and travel time reliability, making high-speed rail a preferred option for intercity commuters [15]. In addition, service quality of transport modes, including safety, comfort, and punctuality, significantly impact travelers’ mode choices by enhancing travel experience utility [16]. This effect is especially critical for commuting trips, for example, stable punctuality and comfortable seating of high- speed rail make it preferred over ordinary trains by intercity commuters.

Besides, network infrastructures and economic factors, like road network accessibility and regional development levels, critically influence modal share by altering the feasibility and cost-effectiveness of transport modes [17]. For example, core cities with developed high-speed rail networks exhibit higher rail modal shares, while underdeveloped regions with limited air or rail infrastructure rely more on highway services. Xu et al. [18] used the passenger transport data during Spring Festival and found that developed eastern coastal cities are dominated by aviation and high-speed rail due to their strong labor force attractiveness, while central labor-exporting regions take highways and conventional railways as the dominant transport modes. These findings indicate that distribution patterns of intercity passenger modal share are significantly correlated with urban economic development and infrastructure completeness degree.

Except for the aforementioned influencing factors, distance serves as a key determinant of modal shares, with the share of each transport mode exhibiting distinct evolution patterns as distance varies. Our prior research [19] identified the mode-specific dominant trip distance ranges for highway, railway, and aviation modes within intercity contexts. For short-distance trips, highway achieves the highest share rate due to its minimal distance constraints, high flexibility, and convenient accessibility. By contrast, for medium- to long-distance trips, railway and aviation keep a considerable level in their modal shares owing to their high-speed advantage. They also experience a notable increase in modal shares over long-distance trips and gradually become dominant choices as distance increases. Furthermore, relevant studies have also confirmed these distance-dependent patterns of modal shares. Gu et al. [20] found that travel distance exerts a significant negative impact on intercity transport volume, with a 1% increase in distance leading to a 1.747% decrease in mobility volume, with varying effect across highway, railway, and aviation modes. Wen et al. [4] revealed that travel distance directly shapes the multimodal model shares: 97.85% of highway flows are concentrated within 500 km, whereas 96.53% of aviation flows occur beyond 500 km. Moreover, from the perspective of the entire travel chain, Li et al. [21] identified travel distance as the dominant factor shaping intercity mode choice: short-distance travel (≤200 km) prefers private transport and intercity buses, medium-distance travel (200–800 km) favors railway and highway, and long-distance travel (>800 km) relies primarily on aviation. This distance-based segmentation is also affected by transfer convenience and travel time reliability, which vary across different transport modes.

Intercity passenger mode share and its influencing factors exhibit remarkable temporal dynamics and spatial heterogeneity [22,23]. In terms of temporal dynamics, seasonal shifts and periodic fluctuations drive significant variations. For instance, international tourists in South Korea showed destination-concentrated travel flows in spring and exhibited dispersed movements in summer, with diurnal peaks in afternoon/evening travel [16].Chu et al. [24] further quantified this periodicity through a study on China’s megalopolises, revealing that intra-megalopolis mobility exhibits an obvious weekly periodicity driven by commuting demand, while inter-megalopolis mobility is dominated by a monthly periodicity. Moreover, Yu et al. [12] and Wen et al. [25] indicated that holidays amplify these dynamics significantly: they not only drive a substantial surge in intercity mobility—particularly during long holidays such as May Day and the combined Mid-Autumn Festival and National Day—but also intensify spatiotemporal fluctuations, with cities classified into inbound, outbound, and balanced types based on population flow directions. Cui et al. [26] found that non-holiday periods exhibit distinct weekly periodicity, with mobility peaks on Mondays and Saturdays dominated by short-distance commuting. In contrast, holidays are characterized by long-distance leisure travel, which becomes the primary driver of intercity mobility flows.

Additionally, spatial heterogeneity is evident across regions and city hierarchies. For example, Gao et al. [27] indicated that U.S. airports in the same region often exhibit similar passenger arrival rhythms, while geographically distant airports serving similar destination types also share analogous patterns. Business-oriented airports tend to exhibit concentrated peaks during weekday mornings, whereas leisure-focused airports display more dispersed or holiday/weekend-centric passenger flows, reflecting how location-driven travel preferences and destination functions shape mobility flow dynamics. In China, highway networks show core-periphery structures, with dense flows in eastern megaregions (e.g., Yangtze River Delta) and sparse connections in western areas, while dual-nuclei (Chengdu-Chongqing) and polycentric (Shanghai-Jiangsu-Zhejiang) structures shape mode share distributions [3]. High-speed rail further exacerbates spatial differences—peripheral cities near high-speed rail lines gain greater accessibility improvements, altering their share relative to highways [17]. It is worth noting that intercity mobility in China presents a diamond-shaped spatial structure anchored by Beijing, Shanghai, Guangzhou-Shenzhen, and Chengdu-Chongqing [4]. This structure is highly consistent with the spatial pattern of labor mobility identified by Xu et al. [18] using Spring Festival passenger transport data, whereby core eastern coastal cities and central cities in central and western China constitute the main flow axis of intercity transportation.

Although existing research has provided a foundation for understanding the dynamic patterns and underlying mechanisms of transport modal shares, substantial limitations persist in the context of intercity passenger transportation systems [28]. First, regarding the scope of research, existing research has largely examined intra-urban commuting flows or mobility flows within core urban agglomerations [18,29], such as the BTH and YRD regions in China, whereas cross-regional passenger flows outside urban agglomerations remains comparatively limited. Moreover, this limited research scope has led to a lack of systematic, nationwide comparisons of intercity passenger modal shares across cities with different development levels and geographical locations. Similarly, in the temporal dimension, existing studies predominantly analyze different periods independently (e.g., weekdays or a specific holiday), lacking comparative analysis across multiple holiday scenarios. Consequently, they also lack an analysis of the multi-temporal scenarios in transportation modal shares [1]. For instance, existing studies rely solely on daily or single-holiday flow data, which cannot reflect the seasonal fluctuations and multi-temporal scenarios of transportation modal shares [28].

Furthermore, the depth of existing research is further constrained by limited data sources. Previous studies have predominantly relied on questionnaires and census records, which inherently suffer from notable shortcomings. For instance, questionnaire-based surveys—such as the stated preference (SP) and revealed preference (RP) surveys employed in Tabasi et al. [30] to estimate willingness-to-pay for travel mode attributes—incur substantial costs and exhibit low response efficiency, often yielding small-scale samples with restricted geographic or corridor-specific coverage. Such limitations render them inadequate for capturing the characteristics of multi-modal and large-scale passenger flows, as they fail to encompass the diverse travel behaviors across extensive spatial domains. On the other hand, census data—exemplified by the 2015 One Percent Population Sample Survey utilized in Mu et al. [31] to explore internal migration patterns along urban hierarchies—are updated infrequently. This infrequency prevents them from reflecting short-term modal shifts triggered by holidays or other transient factors, limiting the understanding of dynamic travel demand fluctuations.

In contrast, location-based service (LBS) data, as demonstrated by Yu et al. [32] using a nationwide dataset to analyze the spatiotemporal dynamics of intercity mobility, can address the afore- mentioned shortcomings of questionnaires and census data with its high spatiotemporal precision, extensive sample coverage, and capacity to capture daily mobility flows [33]. Leveraging its massive user base (for example, encompassing over one billion users in China), LBS data enables accurate characterization of intercity population movement and modal usage, thereby providing robust support for mode share modeling. Mobile device location data represented by Tencent nationwide LBS migration datasets overcomes the inherent limitations of traditional questionnaire and census travel surveys in sample scale and temporal coverage, providing high-frequency positioning records covering over one billion mobile terminals across prefecture-level cities in China [34]. Although emerging data sources such as mobile phone signaling data have been gradually adopted in recent studies—for example, Li et al. [35] utilized such data to quantify the nonlinear and spatial heterogeneous effects of the built environment on intercity commuting—some applications still suffer from insufficient data representativeness. Either they only cover short-term periods or are limited to a subset of cities, failing to capture the long-term, nationwide spatiotemporal dynamics of intercity passenger transportation. Consequently, they cannot effectively support comparative analyses of mode share across different distance ranges and regions. These gaps reveal that intercity passenger mode share research must overcome regional constraints and data limitations, adopt a national-scope data to clarify how distance shapes mode share patterns, and support optimal cross-regional resource allocation.

Drawing upon the aforementioned gaps, this research focuses on the distance-dependent laws of intercity passenger mode shares. Using the long-term large-scale Tencent LBS data from China, which presents the nationwide modal shares of highway, railway, and aviation modes from 2015 to 2018, this study takes travel distance as the key variable to systematically fit nonlinear relationships between multimodal share and travel distance, with comprehensive comparations across socioeconomic and spatiotemporal contexts. The primary contributions of this study are presented as follows:

(1) Examine the spatiotemporal distribution and its dynamic evolution of intercity passenger modal shares for highway, railway, and aviation using the nationwide LBS data.
(2) Establish nonlinear relationships between intercity passenger modal shares and travel distance for highway, railway, and aviation, and develop mode-specific share functions with polynomial fitting of distance.
(3) Conduct a comparative comparison of distance-mode share relationships across socioeconomic scales (economic development level, administrative rank, and population size), and spatiotemporal contexts (weekdays, including weekends, typical holidays, and geographical location).

The remainder of this paper is structured as follows. The Study Area and Data Sources section elaborates on the study area and data sources. The Methodology section outlines the adopted research methodology. The Result Analysis section presents the relationships between travel distance and intercity passenger mode shares, and analyzes the results. The Discussion on Multiscale Comparisons section presents a discussion on the empirical results. Finally, the Conclusions and Future Outlooks section summarizes the key research findings and outlooks future directions.

## Study area and data sources

### Study area

The study area covers 362 cities in mainland China, including directly administered municipalities, prefecture-level cities, and key county-level cities with significant intercity mobility demand. Moreover, this study employs the Tencent LBS data encompassing intercity passenger mode shares of highway, railway, and aviation from 2015 to 2018. This temporal window is primarily determined by data availability in nationwide, long-term, continuous LBS data with consistent mode classification across multiple years, while data after 2018 are not accessible through the platform. Although China’s railway and aviation networks have expanded considerably since 2018, prior research utilizing the same LBS data source has demonstrated that the fundamental structural patterns of intercity passenger mode shares, particularly the distance-dependent relationships, exhibit remarkable stability across years [36]. Moreover, the distance-insensitive characteristics of intercity passenger mode shares are largely determined by the inherent properties of intercity travel modes, such as the speed and cost. As a result, despite infrastructural expansion that may shift specific threshold values, the overarching functional form of the distance–modal share relationship tends to remain robust in China over the past decade, owing to stable mode attributes. Besides, this study period excludes the pandemic and post-pandemic period, which is necessary as COVID-19-related mobility restrictions and lockdown policies severely disrupted regular travel behavior and mode choice over a prolonged period, particularly for aviation, thereby obscuring the natural distance-dependent relationships that this study aims to characterize. This selection aligns with empirical evidence that public health emergencies and associated mobility restrictions drastically reshape intercity travel patterns and modal preferences, especially for long-distance aviation and high-speed rail services [37]. Also, the selected period incorporates special travel peaks such as the Golden Week around National Day and Spring Festival travel rush, enabling the capture of both regular and exceptional mobility patterns. Ultimately, a total of 663,780 valid data records are obtained, encompassing information on origin cities, destination cities, and the modal shares of highway, railway, and aviation. Given the spatiotemporal regions, newly established cities formed after 2019 due to administrative adjustments are excluded, as their mobility data is not available during the data collection period.

### Data sources

The population migration data used in this study are obtained from the Location-Based Services (LBS) provided by Tencent Location Big Data Platform (https://heat.qq.com). As a fundamental component of contemporary mobile ecosystems, LBS delivers location-aware services to users by leveraging their perceived location information—for instance, recommending nearby points of interest, providing accessible travel options, and enabling navigation. In particular, users’ movement trajectories can be collected via high-accuracy Global Positioning System (GPS) technology, with a positioning accuracy of approximately 5–10 meters. Leveraging multiple application scenarios within the Tencent ecosystem, this platform has established a national spatiotemporal monitoring system for population dynamics and can supply the corresponding dataset.

The dataset is constructed based on daily aggregated intercity mobility records obtained from LBS positioning. Travel modes are inferred from anonymized user trajectories via proprietary algorithms, which identify the primary transport mode according to travel speed, trajectory continuity and network path matching. On this basis, the platform publishes mode share percentages for highway, railway, and aviation for each origin-destination (OD) pair. The mode share for a given transport mode is defined as the ratio of passengers travelling via that mode to the total intercity passenger flow across the three modes between the OD pair. The dataset exhibits several advantages, including broad sample coverage, high spatiotemporal resolution, multi-dimensional integration capability, and strong reliability [38–40]. As suggested by Wang et al. [41], passively collected large-scale data enable real-time monitoring and capture temporal dynamics that conventional surveys cannot adequately reflect.

Furthermore, the LBS data has been widely used in existing research in transportation planning and network analysis, with particular applications in the analysis of intercity population flows. For example, Qi et al. [2] employed this dataset to compare the structural characteristics of China’s intercity travel networks across daily, holiday, and Spring Festival travel rush periods, revealing the diamond-shaped core structure centered on Beijing, Shanghai, Guangzhou-Shenzhen, and Chengdu-Chongqing. Gao et al. [27] utilized it to quantify the causal effect of high-speed rail on intercity tourist flows during national holidays, confirming its effectiveness in capturing short-term mobility dynamics. Pan et al. [42] explored the spatial pattern of population mobility during the National Day-Mid-Autumn Festival, identifying distinct community structures in the urban network. Such diverse applications underscore the dataset’s robustness and versatility in advancing geographic and transportation research.

During data acquisition, a custom-developed web crawler coded in Python was used to retrieve the published data. To efficiently manage the collection process, a parameter filtering module was employed to ensure precise screening of records. Specifically, city-level origin–destination (OD) pair parameters were managed via a queued workflow, i.e., only uncollected entries were retrieved, and each processed record was immediately dequeued and used to update the database, thereby preventing duplicate collection. For preprocessing and cleaning, the web-scraped data were first parsed to extract key fields—date, origin and destination cities, and modal shares across highway, railway, and aviation—with the raw responses parsed using HTML and JSON libraries, before being saved locally in JSON format and converted into universal representations, including sequences and tables. The dataset comprises 663,780 valid records, covering 362 cities across China, with data spanning 2015–2018 and including weekdays, weekends, and major holiday periods such as the National Day Golden Week and the Spring Festival travel rush. It is worth noting that the collected data are aggregated and de-identified, containing no personally identifiable information.

Moreover, spherical great-circle distance between city pairs was calculated using urban latitude–longitude coordinates, and then appended to the dataset as the unified distance metric. The choice of this metric is motivated by the inherent discrepancies between highway, railway, and aviation route distances for the same OD pair. Employing a consistent and standardized distance measure is therefore essential to ensure the comparability of subsequent cross-mode analyses. Finally, distance-range validation was performed to identify valid intercity distances within 8–4462 km. Notably, records with travel distances exceeding 3000 km were excluded from this study, because their extremely small sample sizes would inevitably compromise the robustness of subsequent analyses.

As a consequence, the dataset used in this study includes information on date, origin, destination, spherical great-circle distance between city pairs, and modal shares of highway, railway, and aviation. Table 1 presents data samples with the corresponding data formats.

**Table 1:** Dataset of passenger mode share in intercity travel.

Date	Origin	Destination	Modal share of highway	Modal share of railway	Modal share of aviation	Great-circle distance
3 February 2015	Shenyang	Wuhan	11%	26%	63%	1488km
3 February 2015	Shenyang	Shanghai	13%	14%	73%	1188km
……	……	……	……	……	……	……
1 September 2018	Beijing	Wuhan	9%	73%	18%	1051km
1 September 2018	Beijing	Hangzhou	11%	49%	40%	1122km

### Statistical analysis of modal share

**Fig 1:**
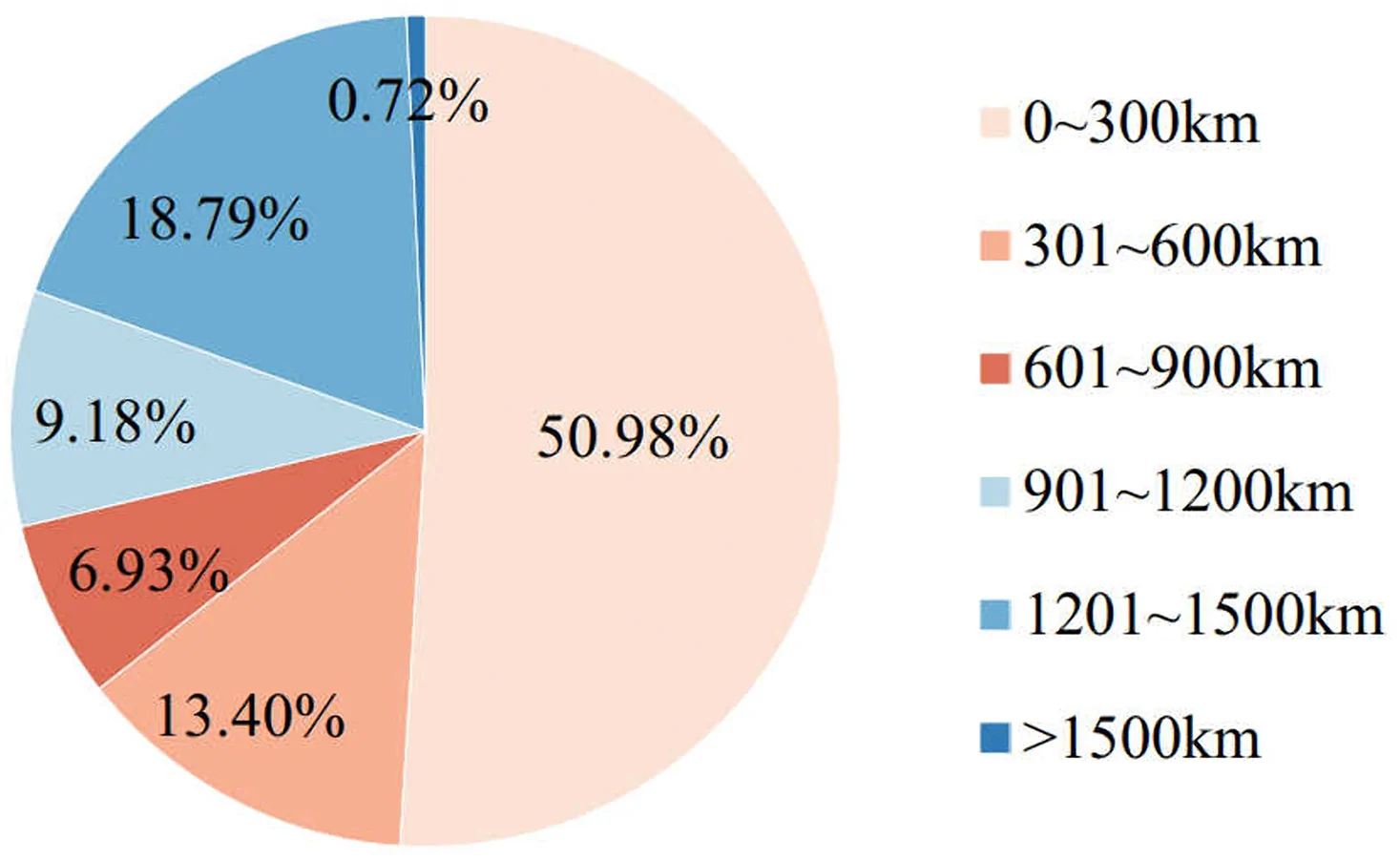
Regional distribution map of passenger travel distances in China.

Travel distance is a pivotal factor influencing passengers’ choice of intercity passenger transport modes. As travel distance increases, travel time and associated economic costs rise correspondingly, while passenger comfort tends to diminish, ultimately shaping intercity passenger modal shares. Given this effect, we first analyzed the distribution of travel distances at a granularity of 300 km. Because the share of travel volume beyond 1500 km is relatively small, all trips with distances greater than 1500 km were grouped into a single category. As shown in Fig 1, intercity passenger travel in China is predominantly concentrated within 300 km, accounting for more than half of the total travel volume, whereas trips over 1500 km account for only 0.72%. Moreover, travel segments of 601–900 km and 901–1500 km constitute a substantial share, suggesting that intercity trips are also strongly represented in the medium-distance range.

**Fig 2:**
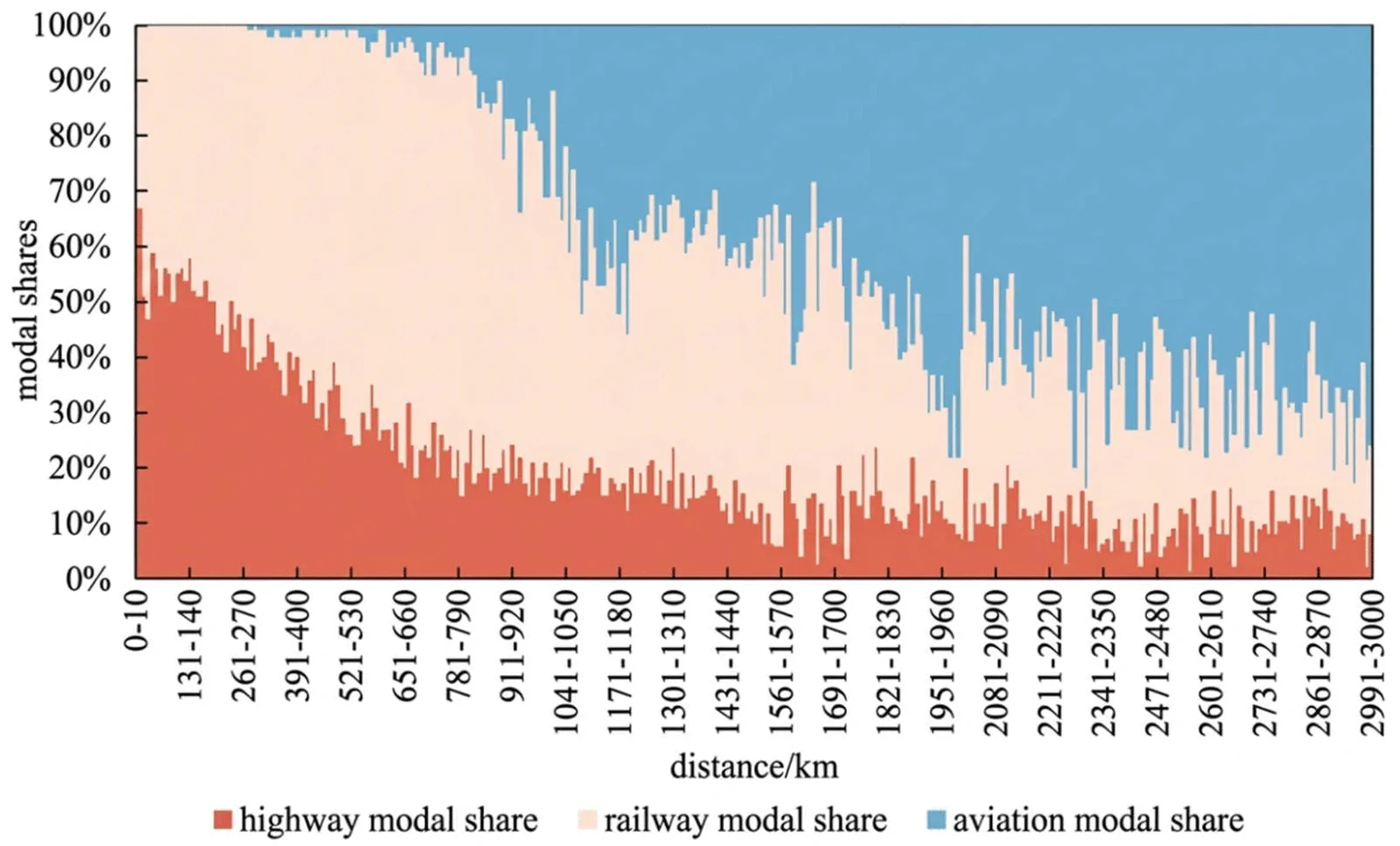
Distribution of intercity passenger transport modal share based on travel distance.

To reflect the distribution patterns more clearly, the entire dataset is further divided at a granularity of 10 km. For each 10-km distance category, the average mode shares of highway, railway, and aviation were computed and then assigned as the modal shares for that category. The modal-share distributions of intercity passenger transport across the travel distance interval of 8–3000 km are presented in Fig 2.

Records exceeding 3,000 km were excluded from this study for two primary reasons. First, intercity travel beyond this threshold is overwhelmingly dominated by aviation, whereas the shares of highway and railway are substantially lower, thereby offering limited analytical value for multimodal share comparison. Second, the number of observations in this extreme tail is extremely sparse, rendering any fitted curve in this range statistically unreliable. In contrast, the 1,500–3,000 km range, while representing only a small proportion of the total observations (0.72%), contains a substantial absolute number of records. This sample size is sufficient to support stable polynomial fitting up to quartic order and exceeds the minimum threshold required for reliable estimation.

As illustrated in the figure, the average highway modal share exceeds 50% for travel distances ranging from 8 to 200 km. It then decreases rapidly as distance increases, falling to below 30% by 900 km. Beyond this point, the downward trend moderates; the highway modal share narrows to within 10% by 3000 km. Over the entire 0–3000 km range, highway consistently remains the transport choice, showing a positive association with shorter travel distances and a negative association with longer ones. Rail transport accounts for a substantial share of intercity trips across the 8–3000 km distance interval. For trips within 30 km, the railway modal share is approximately 30%. It rises to over 40% on average for the 30–200 km range, and reaches around 50% for 200–1000 km. Moreover, railway modal share concentrates at 40%–60% for trips of 1000–1600 km. Afterward, it declines gradually as distance increases, yet remains within 10%–25% even in the 2500–3000 km interval. Aviation modal share is extremely low (below 2%) for travel distances under 500 km, and then increases steadily as distance grows. A substantial proportion of passengers choose aviation for trips over 1500 km, implying that the likelihood of selecting aviation rises with longer travel distances.

Function-fitting results reveal that for short-distance trips highway dominates mode share (>50%), but its share falls off as travel distance increases. By contrast, rail’s modal share stays substantial through medium-to-long ranges, peaking in the 1,000–1,900 km band. This reflects HSR’s competitive edge in those corridors. Indeed, a recent analysis identifies ~1,160 km as the break-even distance for HSR versus air travel [43]. In our fitted curves, aviation share is minimal below ~800 km and only rises for the extremely long journeys, suggesting that the access delays and processing time of airports (ground transport, check-in, security) erode the aviation’s advantage in mid-distance markets. In these mid-range segments, HSR’s advantages, such as city-center station access, frequent services, high on-time performance, and comfortable, secure travel, allow it to capture market share. For example, user satisfaction with HSR’s affordability and convenience strongly boosts its mode share [44]. By contrast, airline services are relatively less frequent. On major city pairs such as Beijing–Shanghai, hundreds of high-speed rail (HSR) services operate in parallel with a substantially smaller number of flights, highlighting the intense intermodal competition on these corridors [45]. This pattern suggests that HSR has captured a dominant share of medium- distance travel demand. Accordingly, policy efforts should prioritize the continued enhancement of HSR—such as improving feeder connectivity and increasing service frequency—while airlines may consider reallocating capacity toward longer-haul markets.

## Methodology

### Research problem definition

In intercity transportation systems, the modal share of different travel modes can be viewed as the equilibrium outcome of a dynamic choice process in which travelers evaluate generalized utility. This utility is jointly shaped by time cost, monetary cost, and service-related attributes, such as comfort and convenience. Travelers are assumed to select the mode that maximizes their perceived utility. Within this framework, travel distance acts as a fundamental determinant of the underlying utility components, and therefore is expected to exert a systematic influence on the distribution of intercity modal shares across transport modes. Accordingly, developing distance-based models for multimodal modal-share is not only theoretically meaningful, but also practically relevant for uncovering the mechanism underlying travelers’ intercity mode choices.

Building upon this perspective, this study aims to investigate the relationship between intercity passenger modal shares and travel distance, and to examine how this relationship varies across socioeconomic and spatiotemporal contexts. Specifically, the intercity travel distance, measured as the great-circle distance between each origin–destination pair, is employed as the independent variable using a consistent metric across all observations. The corresponding modal shares of highway, railway, and aviation serve as the dependent variables. As a result, this study establishes distance-dependent functions for modal shares of highway, railway, and aviation, and derives the corresponding polynomial curves.

### Curve fitting method

In this study, polynomial fitting is adopted to establish the relationship curve between intercity passenger modal shares and travel distance. Compared with simple linear specifications, polynomial fitting provides a flexible functional form that can capture potential non-linearities and curvature in the distance–modal share relationship. It allows modal share distribution to vary smoothly across different distance ranges rather than assuming a constant marginal effect of distance. Moreover, polynomial fitting enables an interpretable characterization of how highway, railway, and aviation shares evolve over distance, which facilitates comparison of the distance-dependent patterns among modes [19].

The parameters of the polynomial functions are estimated using the least-squares method, which is widely applied in curve fitting. Given a dataset that records the modal share of a specific transport mode across different distance, denote as $D=\{(x_{\text{1}},y_{\text{1}}),(x_{\text{2}},y_{\text{2}}),(x_{\text{3}},y_{\text{3}}),\ldots \;,(x_{n},y_{n})\}$. The research object is to establish f(x) as $f\left(x\right)=a_{\text{0}}+a_{\text{1}}x+{a_{\text{2}}x}^{\text{2}}+\ldots +{a_{k}x}^{k}$. The principle of polynomial fitting function f(x) by the method of least square methods is minimizing the sum of square variance between the observed modal shares and the fitted values, which is denoted as *Q*:


$$\begin{array}{c} \text{Q}=\sum_{i=\text{1}}^{n}{\left({\text{y}}_{\text{i}}-[{\text{a}}_{\text{0}}+{\text{a}}_{\text{1}}{\text{x}}_{\text{i}}+{\text{a}}_{\text{2}}{\text{x}}_{\text{i}}^{\text{2}}+\ldots +{\text{a}}_{\text{k}}{\text{x}}_{\text{i}}^{\text{k}}]\right)}^{\text{2}} \end{array}$$ (1)

To solve the optimal solution of Q, the problem is transformed into setting the partial derivatives with respect to each polynomial coefficients to 0. Solving a system of linear equations allows us to solve the optimal solution of partial derivatives, as shown in Equation (2).


$$\begin{array}{c} \begin{array}{c} \text{n}{\text{a}}_{\text{0}}+{\text{a}}_{\text{1}}\sum_{\text{i}=\text{1}}^{\text{n}}{\text{x}}_{\text{i}}+{\text{a}}_{\text{2}}\sum_{\text{i}=\text{1}}^{\text{n}}{{\text{x}}_{\text{i}}}^{\text{2}}+\ldots +{\text{a}}_{\text{k}}\sum_{\text{i}=\text{1}}^{\text{n}}{{\text{x}}_{\text{i}}}^{\text{k}}=\sum_{\text{i}=\text{1}}^{\text{n}}{\text{y}}_{\text{i}}\;\\ {\text{a}}_{\text{0}}\sum_{\text{i}=\text{1}}^{\text{n}}{\text{x}}_{\text{i}}+{\text{a}}_{\text{1}}\sum_{\text{i}=\text{1}}^{\text{n}}{{\text{x}}_{\text{i}}}^{\text{2}}+{\text{a}}_{\text{2}}\sum_{\text{i}=\text{1}}^{\text{n}}{{\text{x}}_{\text{i}}}^{\text{3}}+\ldots +{\text{a}}_{\text{k}}\sum_{\text{i}=\text{1}}^{\text{n}}{{\text{x}}_{\text{i}}}^{\text{k}+\text{1}}=\sum_{\text{i}=\text{1}}^{\text{n}}{{\text{x}}_{\text{i}}\text{y}}_{\text{i}}\\ \begin{array}{c} \ldots \ldots \\ {\text{a}}_{\text{0}}\sum_{\text{i}=\text{1}}^{\text{n}}{{\text{x}}_{\text{i}}}^{\text{k}}+{\text{a}}_{\text{1}}\sum_{\text{i}=\text{1}}^{\text{n}}{{\text{x}}_{\text{i}}}^{\text{k}+\text{1}}+{\text{a}}_{\text{2}}\sum_{\text{i}=\text{1}}^{\text{n}}{{\text{x}}_{\text{i}}}^{\text{k}+\text{2}}+\ldots +{\text{a}}_{\text{k}}\sum_{\text{i}=\text{1}}^{\text{n}}{{\text{x}}_{\text{i}}}^{\text{2k}}=\sum_{\text{i}=\text{1}}^{\text{n}}{{{\text{x}}_{\text{i}}}^{\text{k}}\text{y}}_{\text{i}} \end{array} \end{array} \end{array}$$ (2)

To evaluate the goodness of the fit, the adjusted R-squared (R^2^) was calculated [19]. Mathematically, the adjusted R-squared is written in Equation (3), where $\overset{―}{y}$ represents the average value, $\hat{y_{i}}$ indicates the fitted value, $y_{i}$ is the real value, n represents the number of samples, and p is the number of explanatory variables.


$$\begin{array}{c} {R_{adjusted}}^{\text{2}}=\text{1}-\frac{n-\text{1}}{n-p-\text{1}}\left[\text{1}-\frac{\sum_{i=\text{1}}^{n}{(\hat{y_{i}}-\overset{―}{y})}^{\text{2}}}{\sum_{i=\text{1}}^{n}{(y_{i}-\overset{―}{y})}^{\text{2}}}\right] \end{array}$$ (3)

It should be emphasized that the adjusted R^2^ is employed in this study primarily as a descriptive measure of goodness-of-fit for the distance-modal share curves, rather than as a validation of predictive performance. The consistently high adjusted R^2^ values indicate that the polynomial functions capture the dominant nonlinear patterns in the distance–modal share relationship across the investigated contexts, thereby supporting the descriptive and comparative analyses that constitute the core of this study.

### Comparison framework

This study conducts a comprehensive comparison regarding the distance-dependent modal shares of highway, railway, and aviation across different socioeconomic and spatiotemporal contexts. To be more specific, socioeconomic aspect groups cities based on their economic scale, administrative rank, and population scale. From a spatiotemporal perspective, this study considers different temporal periods, including seasons, festivals (the Spring Festival and Golden Week), weekdays, and weekends, and accounts for the cities’ geographical location. The comparison framework of intercity passenger modal shares is summarized in Table 2. It should be noted that, in this study, socioeconomic attributes serve only as grouping criteria of cities rather than regression covariates. Accordingly, we focus on characterizing the distance‑dependent patterns of intercity modal share via stratified comparative analysis, and we do not aim to build a comprehensive predictive or causal‑inference model incorporating all potential confounding factors.

**Table 2:** Spatiotemporal comparison framework of intercity passenger modal shares.

Dimensionality	Category	Specific time and space range
Temporal heterogeneity	Whether it is a weekday or not	Weekdays, Weekends
	Whether it is Golden Week or not	Golden Week, Non-Golden Week
	Whether it is Spring Festival travel rush or not	Spring Festival travel rush, non-Spring Festival travel rush
	Seasonal classification	Spring, Summer, Autumn, Winter
Spatial heterogeneity	Geographical location	The eastern coastal region, The southern coastal region, The northern coastal region, The middle reaches of the Yangtze River Region, The northwestern region, The northeast Region, The middle reaches of the Yellow River Region, The southwest region
Socioeconomic heterogeneity	Economic development level	First-tier cities, Second-tier cities, Third-tier cities, Fourth-tier cities, Fifth-tier cities
	Administrative rank	Provincial-level cities, Sub-provincial-level cities, Prefecture-level cities, County-level cities
	Population size	Megacities, Super cities, Large cities, Medium-sized cities, Small cities

## Result analysis

### Modal share curves upon distance

**Fig 3:**
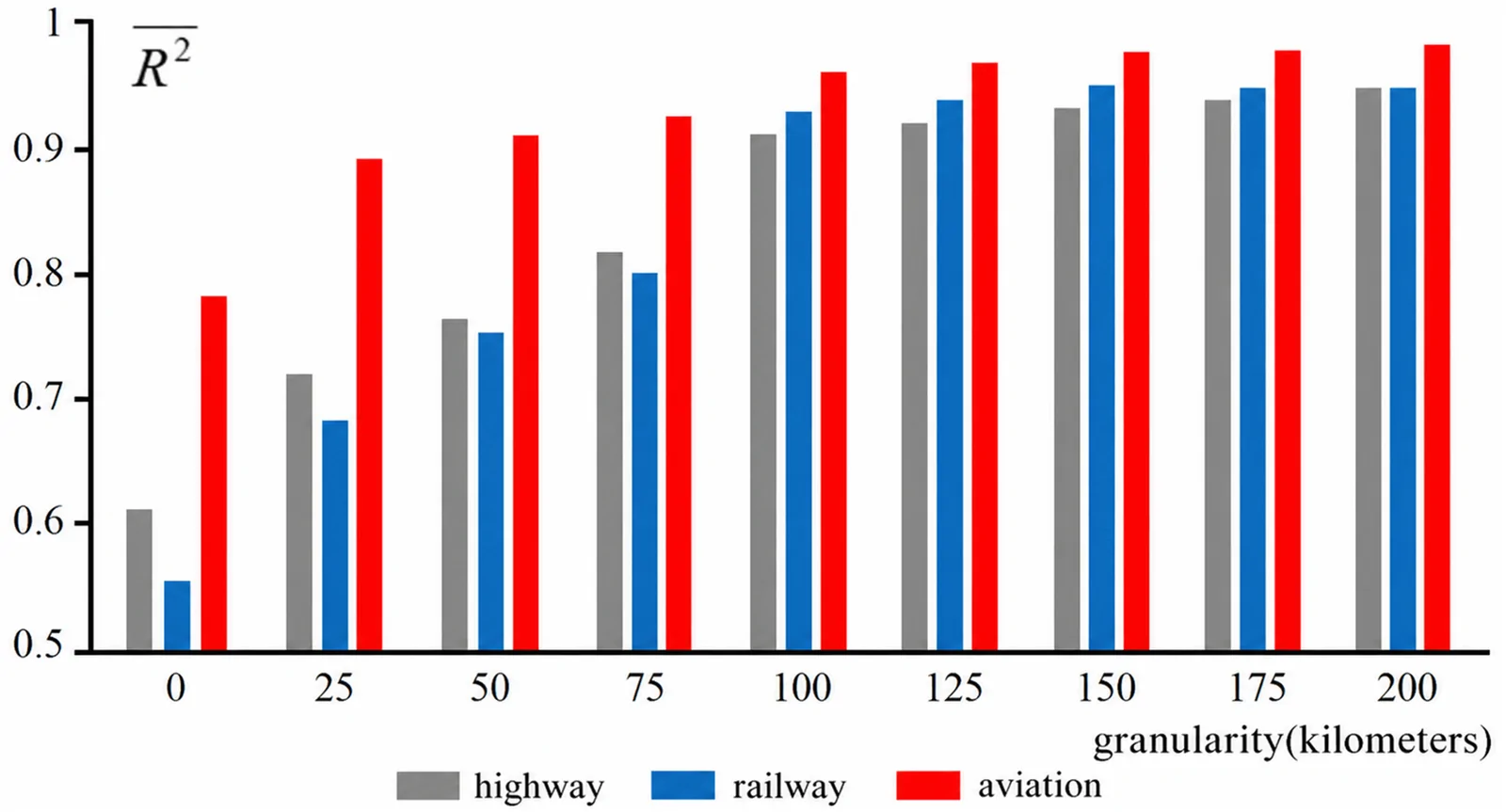
The results of the adjusted R-squared with different particle sizes.

Given the large sample size, categorical analysis was applied to mitigate the influence of noise. Following the methodological framework proposed by our prior research [19], this study adopts a 100 km interval to aggregate intercity travel data. This granularity was selected through a sensitivity experiment, as shown in Fig 3. Specifically, we evaluated the adjusted R^2^ values of polynomial fits for highway, railway, and aviation modal shares across aggregation granularities ranging from 25 km to 200 km at 25 km increments. The results demonstrate that the adjusted R^2^ values for all three modes stabilized beyond the 100 km threshold, consistently exceeding 0.9, whereas smaller intervals yield noisier estimation results owing to limited sample sizes. On this basis, it should be emphasized that the 10 km aggregation presented in Fig 2 is employed solely for descriptive visualization of the distribution patterns, rather than serving as the basis for curve fitting.

Accordingly, in the polynomial fitting procedure, for each 100 km bin, modal shares of highway, railway, and aviation are computed as the arithmetic means of all observations within that interval and assigned to the median travel distance of that bin. Preliminary statistical screening across all 100-km distance bins indicates that modal share values of all OD pairs within the same bin fluctuate within a relatively narrow and comparable range. This concentrated distribution characteristic ensures that, when extracting the general distance-dependent modal share patterns that are the primary focus of this study, the use of arithmetic means does not introduce substantial bias. Based on this aggregation scheme, we derive the fitted distance–modal share curves for the three transport modes across various socioeconomic and spatiotemporal contexts, and conduct a systematic comparative analysis.

The polynomial order for each mode and subgroup is determined through a systematic forward-selection procedure. Starting from a linear (first-order) specification, we incrementally increase the polynomial degree and compute the corresponding adjusted R^2^ for each candidate model. The selection criterion balances goodness-of-fit against model parsimony: a higher-order polynomial is retained only if it yields an improvement in adjusted R^2^ of at least 0.01 over the lower-order alternative; otherwise, the more parsimonious specification is preferred. This procedure is applied separately to each mode and subgroup, allowing for mode-specific functional forms that best capture the underlying distance–share relationship. It should be noted that the selected polynomial degrees vary across subgroups, ranging from cubic to quartic polynomials, reflecting the intrinsic characteristics of distinct modal share curves instead of arbitrary model selection. The consistently high adjusted R^2^ values (all above 0.9) indicate that the polynomial functions adequately capture the dominant nonlinear patterns in the data.

For aviation modal share, a piecewise specification is adopted to account for the near-absence of air travel over short distances. Specifically, we first examine the empirical distribution of aviation shares across distance bins and identify the threshold below which the observed aviation share remains consistently below 2%. This distance threshold, varying across subgroups from approximately 230 km to 490 km, is then set as the breakpoint, below which the aviation is considered a negligible alternative mode and its modal share is fixed at zero, and above which a polynomial function is fitted to the observed shares. Such a treatment is empirically grounded and prevents the polynomial from producing implausible positive predictions at extremely short distances.

### Comparative analysis in temporal perspective

To analyze the temporal heterogeneity in intercity passenger modal shares, we categorize the travel periods into: (i) Seasonal classification: spring, summer, autumn, and winter; (ii) Workday classification: weekdays and weekends; (iii) Golden Week classification: Golden Week versus non-Golden Week; (iv) Spring Festival classification: Spring Festival travel rush versus non-Spring Festival travel rush.

#### Analysis of temporal heterogeneity in highway modal share.

**Fig 4:**
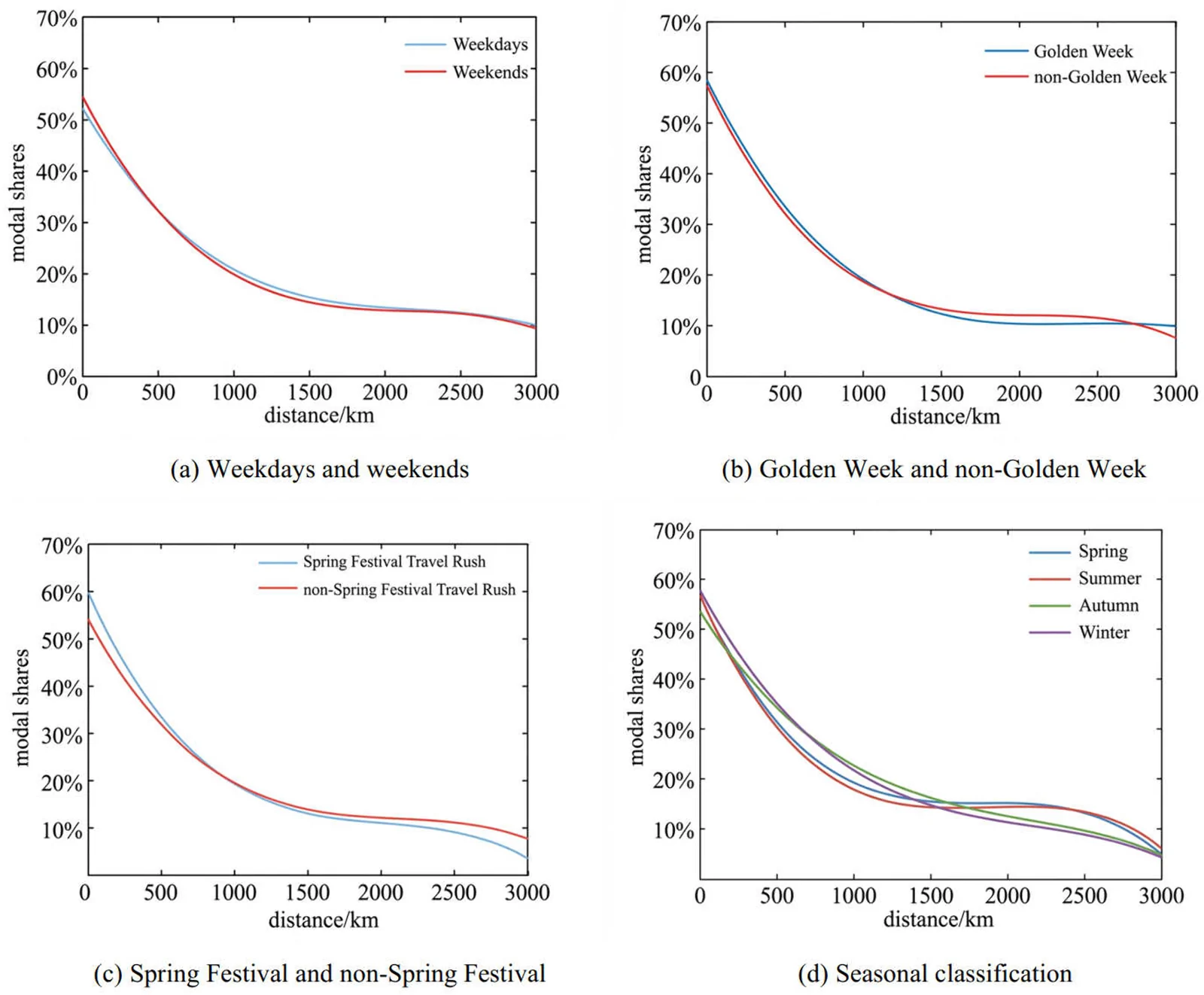
The comparative analysis of highway modal shares across temporal contexts.

As shown in Fig 4 and Table 3, the travel periods have a certain influence on highway modal shares. Specifically, different seasons and whether trips occur during the Spring Festival travel rush exert a relatively strong influence, whereas trips made on weekdays or weekends show a comparatively smaller impact.

**Table 3:** Fitting curves of highway modal shares across temporal contexts.

Study periods		Distance-based fitting curves of highway modal shares	R^2^
(i)	Weekdays	$f_{\text{1}}\left(x\right)=-3.429\times 10^{-9}x^{\text{3}}+2.252\times 10^{-5}x^{\text{2}}-5.104\times 10^{-2}x+52.67$	0.931
	Weekends	$f_{\text{1}}\left(x\right)=-4.059\times 10^{-9}x^{\text{3}}+2.604\times 10^{-5}x^{\text{2}}-5.68\times 10^{-2}x+54.68$	0.951
(ii)	Golden Week	$f_{\text{1}}\left(x\right)=-3.744\times 10^{-9}x^{\text{3}}+2.652\times 10^{-5}x^{\text{2}}-6.21\times 10^{-2}x+58.35$	0.999
	Non-Golden Week	$f_{\text{1}}\left(x\right)=-5.017\times 10^{-9}x^{\text{3}}+3.107\times 10^{-5}x^{\text{2}}-6.469\times 10^{-2}x+57.25$	0.999
(iii)	Spring Festival travel rush	$f_{\text{1}}\left(x\right)=-5.222\times 10^{-9}x^{\text{3}}+3.172\times 10^{-5}x^{\text{2}}-6.685\times 10^{-2}x+59.71$	0.998
	non-Spring Festival travel rush	$f_{\text{1}}\left(x\right)=-4.039\times 10^{-9}x^{\text{3}}+2.573\times 10^{-5}x^{\text{2}}-5.629\times 10^{-2}x+54.18$	0.999
(iv)	Spring	$f_{\text{1}}\left(x\right)=-6.555\times 10^{-9}x^{\text{3}}+3.635\times 10^{-5}x^{\text{2}}-6.739\times 10^{-2}x+56.8$	0.999
	Summer	$f_{\text{1}}\left(x\right)=-6.684\times 10^{-9}x^{\text{3}}+3.779\times 10^{-5}x^{\text{2}}-7.008\times 10^{-2}x+56.77$	0.998
	Autumn	$f_{\text{1}}\left(x\right)=-3.009\times 10^{-9}x^{\text{3}}+1.934\times 10^{-5}x^{\text{2}}-4.709\times 10^{-2}x+53.37$	0.999
	Winter	$f_{\text{1}}\left(x\right)=-3.705\times 10^{-9}x^{\text{3}}+2.393\times 10^{-5}x^{\text{2}}-5.629\times 10^{-2}x+57.76$	0.999

As depicted in Fig 4(a), the highway modal share curves for weekday and weekend trips display minimal discrepancies. For travel distances within approximately 450 km, the weekend highway modal share is slightly higher than that of weekdays; beyond this threshold, the weekday highway modal share is marginally greater. Fig 4(b) reveals that for trips shorter than around 1100 km, the proportion of travelers opting for highway transport during Golden Week exceeds that in non-Golden Week periods; for longer distances, however, travelers generally show a stronger preference for highway mode in non-Golden Week. This pattern can be attributed to two key factors. First, the highway toll exemption policy during Golden Week incentivizes short‑to‑medium‑distance self‑driving trips, which dominate holiday leisure travel. Second, for long‑distance journeys, severe highway congestion and limited time efficiency during peak holidays make high‑speed rail and air travel more competitive, whereas non‑holiday conditions offer more stable highway service and greater flexibility for business and routine trips. According to Fig 4(c), the highway modal share during the Spring Festival Travel Rush (hereafter referred to as SFTR) is higher than that in non-SFTR periods for trips under 900 km but lower for longer-distance trips.

As shown in Fig 4(d), the highway modal share curves for spring and summer are analogous, while those for autumn and winter are similar. For trips within 1500 km, the highway modal share in autumn and winter is generally higher than that in spring and summer, likely because cold weather encourages travelers to choose convenient self-driving to avoid transfers and connections. For trips exceeding 1500 km, the proportion of highway travel in spring and summer is overall higher than that in autumn and winter, primarily as adverse weather conditions in autumn and winter compromise road safety for self-driving travel. For trips under 650 km, winter records the highest highway modal share, which may be attributed to the SFTR falling within winter when short-to-medium-distance trips exhibit a marked preference for highway travel, thereby elevating the overall winter modal share.

#### Analysis of temporal heterogeneity in railway modal share.

**Fig 5:**
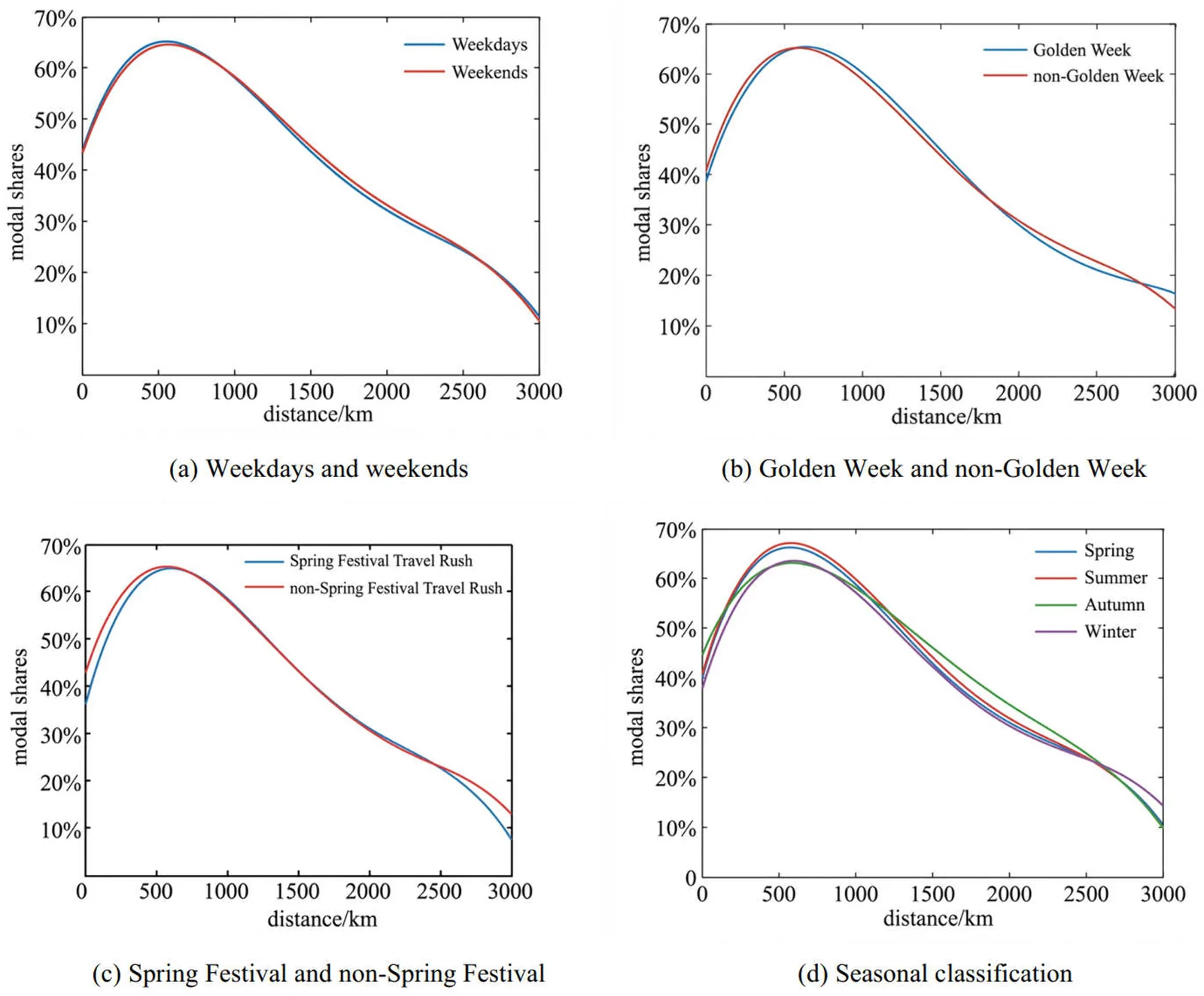
Comparative analysis of railway modal shares across temporal contexts.

As illustrated in Fig 5 and Table 4, the travel periods have a certain influence on railway modal shares. Specifically, seasonal variations and whether trips occur during the SFTR exerts a relatively more pronounced influence on the railway modal share, while whether trips occur on weekdays or during Golden Week has a relatively weaker impact on the highway modal share.

**Table 4:** Fitting curves of railway modal shares across temporal contexts.

Study periods		Distance-based fitting curves of railway modal shares	R^2^
(i)	Weekdays	$f_{\text{2}}\left(x\right)=-6.461\times 10^{-12}x^{\text{4}}+4.638\times 10^{-8}x^{\text{3}}-1.142\times 10^{-4}x^{\text{2}}+8.889\times 10^{-2}x+43.42$	0.943
	Weekends	$f_{\text{2}}\left(x\right)=-6.184\times 10^{-12}x^{\text{4}}+4.43\times 10^{-8}x^{\text{3}}-1.1\times 10^{-4}x^{\text{2}}+8.747\times 10^{-2}x+42.7$	0.948
(ii)	Golden Week	$f_{\text{2}}\left(x\right)=-4.33\times 10^{-12}x^{\text{4}}+3.741\times 10^{-8}x^{\text{3}}-1.079\times 10^{-4}x^{\text{2}}+9.667\times 10^{-2}x+38.34$	0.989
	Non-Golden Week	$f_{\text{2}}\left(x\right)=-5.806\times 10^{-12}x^{\text{4}}+4.433\times 10^{-8}x^{\text{3}}-1.156\times 10^{-4}x^{\text{2}}+9.556\times 10^{-2}x+40.42$	0.996
(iii)	Spring Festival travel rush	$f_{\text{2}}\left(x\right)=-7.823\times 10^{-12}x^{\text{4}}+5.601\times 10^{-8}x^{\text{3}}-1.385\times 10^{-4}x^{\text{2}}+0.1133x+35.47$	0.993
	non-Spring Festival travel rush	$f_{\text{2}}\left(x\right)=-6.039\times 10^{-12}x^{\text{4}}+4.516\times 10^{-8}x^{\text{3}}-1.15\times 10^{-4}x^{\text{2}}+9.183\times 10^{-2}x+42.34$	0.984
(iv)	Spring	$f_{\text{2}}\left(x\right)=-7.842\times 10^{-12}x^{\text{4}}+5.587\times 10^{-8}x^{\text{3}}-1.359\times 10^{-4}x^{\text{2}}+0.1066x+39.87$	0.993
	Summer	$f_{\text{2}}\left(x\right)=-7.729\times 10^{-12}x^{\text{4}}+5.517\times 10^{-8}x^{\text{3}}-1.35\times 10^{-4}x^{\text{2}}+0.1068x+40.44$	0.990
	Autumn	$f_{\text{2}}\left(x\right)=-4.909\times 10^{-12}x^{\text{4}}+3.537\times 10^{-8}x^{\text{3}}-9.03\times 10^{-5}x^{\text{2}}+7.356\times 10^{-2}x+44.25$	0.991
	Winter	$f_{\text{2}}\left(x\right)=-6.71\times 10^{-12}x^{\text{4}}+4.973\times 10^{-8}x^{\text{3}}-1.254\times 10^{-4}x^{\text{2}}+0.102x+37.44$	0.994

As shown in Fig 5(a) and 5(b), the differences in railway modal share between weekend and weekdays, golden week and non-golden week are relatively slight. For travel distances less than approximately 600 km, the proportion of choosing railway during Golden Week is lower than that in non-Golden Week periods; for distances under around 900 km, the weekend railway modal share is lower than that on weekdays. Fig 5(c) reveals that when travel distance exceeds about 600 km, the railway modal share during the SFTR is apparently smaller than that in non-SFTR periods. This may be attributed to two factors: On the one hand, during SDTR, the exemption of highway tolls substantially reduces highway travel costs. On the other hand, during SFTR, the sharp increase in demand for railway tickets leads to supply–demand imbalance, making it difficult for some travelers to obtain tickets and prompting them to switch to other travel options. In summary, for short-to-medium-distance trips, the proportion of railway travel during holidays is lower than in regular periods. When travel distance exceeds approximately 2500 km, the proportion of choosing railway during SFTR is notably lower than usual. This may be also attributed to the supply–demand imbalance of railway tickets during this period.

According to Fig 5(d), the railway modal share curves for spring and summer are similar, as are those for autumn and winter. For distances ranging from 1200 to 2600 km, autumn’s railway modal share is observably higher than that of other seasons. When distance exceeds 2600 km, winter’s railway modal share is apparently greater than that in other seasons. When distance exceeds 2600 km, winter’s railway modal share is notably greater than in other seasons. Overall, for distances within 1000 km, the proportion of choosing railway in spring and summer is higher than that in autumn and winter; for distances over 1000 km, the railway modal share in summer and winter is roughly the same, and apparently lower than that in spring. It can be observed that when travel distance is less than approximately 1200 km, the railway modal share exhibits a strong interaction and negative correlation with the highway modal share across the four seasons.

#### Analysis of temporal heterogeneity in aviation modal share.

**Fig 6:**
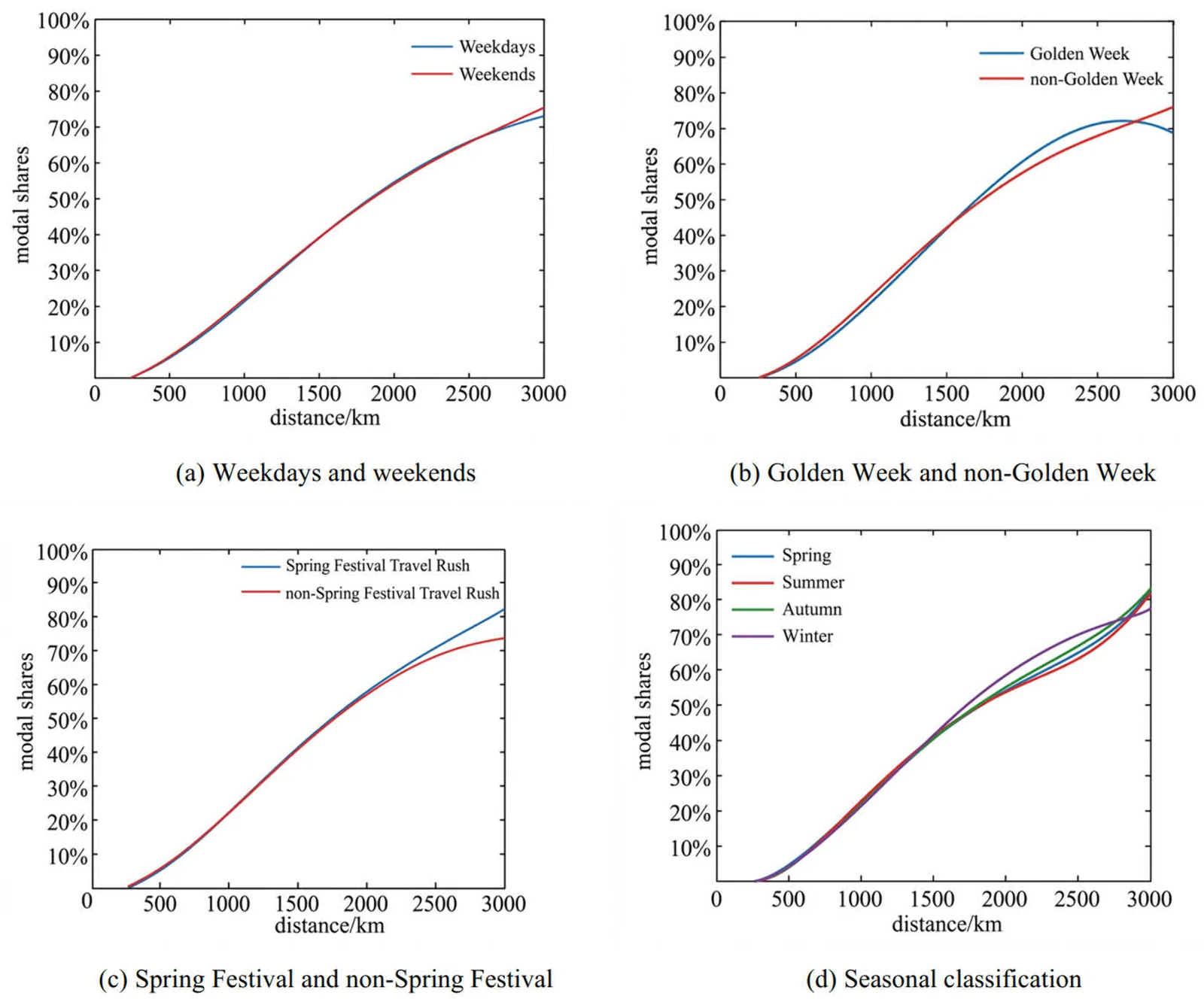
Comparative analysis of aviation modal shares across temporal contexts.

As illustrated in Fig 6 and Table 5, the impact of travel periods on aviation modal share is primarily manifested in long-distance and ultra-long-distance trips. Specifically, seasonal variations and whether trips occur during the SFTR exert a relatively pronounced influence on aviation modal share.

**Table 5:** Fitting curves of aviation modal shares across temporal contexts.

Study periods		Distance-based fitting curves of aviation modal shares	R^2^
(i)	Weekdays	$\left\{ \begin{array}{c} @l@f_{\text{3}}\left(x\right)=0\;\left(\text{8}\leq x<250\right)\\ f_{\text{3}}\left(x\right)=1.421\times 10^{-12}x^{\text{4}}-1.27\times 10^{-8}x^{\text{3}}+3.321\times 10^{-5}x^{\text{2}}+1.286\times 10^{-3}x-2.109\;\left(250\leq x\leq 3\text{000}\right) \end{array}\right.$	0.987
	Weekends	$\left\{ \begin{array}{c} @l@f_{\text{3}}\left(x\right)=0\;\left(\text{8}\leq x<250\right)\\ f_{\text{3}}\left(x\right)=1.63\times 10^{-12}x^{\text{4}}-1.29\times 10^{-8}x^{\text{3}}+3.112\times 10^{-5}x^{\text{2}}+4.705\times 10^{-3}x-2.83\;\left(250\leq x\leq 3\text{000}\right) \end{array}\right.$	0.991
(ii)	Golden Week	$\left\{ \begin{array}{c} @l@f_{\text{3}}\left(x\right)=0\;\left(\text{8}\leq x<280\right)\\ f_{\text{3}}\left(x\right)=2.81\times 10^{-13}x^{\text{4}}-9.779\times 10^{-9}x^{\text{3}}+3.613\times 10^{-5}x^{\text{2}}-4.691\times 10^{-3}x-1.287\;\left(280\leq x\leq 3\text{000}\right) \end{array}\right.$	0.997
	Non-Golden Week	$\left\{ \begin{array}{c} @l@f_{\text{3}}\left(x\right)=0\;\left(\text{8}\leq x<270\right)\\ f_{\text{3}}\left(x\right)=2.685\times 10^{-12}x^{\text{4}}-2.058\times 10^{-8}x^{\text{3}}+4.835\times 10^{-5}x^{\text{2}}-6.612\times 10^{-3}x-1.434\;\left(270\leq x\leq 3\text{000}\right) \end{array}\right.$	0.996
(iii)	Spring Festival travel rush	$\left\{ \begin{array}{c} @l@f_{\text{3}}\left(x\right)=0\;\left(\text{8}\leq x<270\right)\\ f_{\text{3}}\left(x\right)=2.229\times 10^{-12}x^{\text{4}}-1.72\times 10^{-8}x^{\text{3}}+4.19\times 10^{-5}x^{\text{2}}-3.027\times 10^{-3}x-1.851\;\left(270\leq x\leq 3\text{000}\right) \end{array}\right.$	0.995
	non-Spring Festival travel rush	$\left\{ \begin{array}{c} @l@f_{\text{3}}\left(x\right)=0\;\left(\text{8}\leq x<250\right)\\ f_{\text{3}}\left(x\right)=1.243\times 10^{-12}x^{\text{4}}-1.233\times 10^{-8}x^{\text{3}}+3.366\times 10^{-5}x^{\text{2}}+1.707\times 10^{-3}x-2.254\;\left(250\leq x\leq 3\text{000}\right) \end{array}\right.$	0.994
(iv)	Spring	$\left\{ \begin{array}{c} @l@f_{\text{3}}\left(x\right)=0\;\left(\text{8}\leq x<290\right)\\ f_{\text{3}}\left(x\right)=4.462\times 10^{-12}x^{\text{4}}-2.901\times 10^{-8}x^{\text{3}}+6.028\times 10^{-5}x^{\text{2}}-1.288\times 10^{-2}x-0.7562\;\left(290\leq x\leq 3\text{000}\right) \end{array}\right.$	0.995
	Summer	$\left\{ \begin{array}{c} @l@f_{\text{3}}\left(x\right)=0\;\left(\text{8}\leq x<320\right)\\ f_{\text{3}}\left(x\right)=6.086\times 10^{-12}x^{\text{4}}-3.859\times 10^{-8}x^{\text{3}}+7.794\times 10^{-5}x^{\text{2}}-2.375\times 10^{-2}x+0.7165\;\left(320\leq x\leq 3\text{000}\right) \end{array}\right.$	0.996
	Autumn	$\left\{ \begin{array}{c} @l@f_{\text{3}}\left(x\right)=0\;\left(\text{8}\leq x<320\right)\\ f_{\text{3}}\left(x\right)=4.407\times 10^{-12}x^{\text{4}}-2.954\times 10^{-8}x^{\text{3}}+6.41\times 10^{-5}x^{\text{2}}-1.787\times 10^{-2}x+0.184\;\left(320\leq x\leq 3\text{000}\right) \end{array}\right.$	0.994
	Winter	$\left\{ \begin{array}{c} @l@f_{\text{3}}\left(x\right)=0\;\left(\text{8}\leq x<290\right)\\ f_{\text{3}}\left(x\right)=2.337\times 10^{-12}x^{\text{4}}-1.976\times 10^{-8}x^{\text{3}}+5.073\times 10^{-5}x^{\text{2}}-1.169\times 10^{-2}x-0.4381\;\left(290\leq x\leq 3\text{000}\right) \end{array}\right.$	0.995

As shown in Fig 6(a), the aviation modal share curves for weekday and weekend trips exhibit minimal discrepancies. Fig 6(b) reveals that for travel distances less than 1500 km, the aviation modal share during Golden Week is lower than that in non-Golden Week periods; for distances ranging from 1500 to 2750 km, travelers are more likely to choose aviation during Golden Week compared to regular periods. This can be attributed to the fact that Golden Week lasts only seven days—travelers tend to prioritize shorter travel time to maximize leisure, tourism, or family visit opportunities, thus preferring aviation for long and ultra-long-distance trips due to its high speed and short journey time, which is consistent with the railway modal share curves during Golden Week. When travel distance reaches approximately 2500 km, the aviation modal share begins to decline; this anomaly may stem from the small sample size of trips exceeding 2500 km during Golden Week, leading to substantial data fluctuations. According to Fig 6(c), for travel distances greater than 1500 km, the aviation modal share during the SFTR consistently exceeds that in non-SFTR periods. This is driven by the surge in travel demand during the Spring Festival and the aviation’s advantage of short travel time, resulting in a significant increase in the proportion of aviation for long and ultra-long-distance trips.

As illustrated in Fig 6(d), for travel distances less than 1500 km, there is no apparent difference in aviation modal share across four seasons; for distances greater than 1500 km, the proportion of aviation in winter is generally observably higher than that in other seasons. This indicates that travelers are more inclined to choose aviation for long and ultra-long-distance trips in winter, likely attributed to reduced choice on highway in winter due to cold weather and slippery road conditions. Additionally, the aviation modal share trends for spring, summer, and autumn are highly similar: for distances ranging from 1700 to 3000 km, autumn records the highest aviation modal share, followed by spring and summer.

### Comparative analysis in spatial perspective

**Fig 7:**
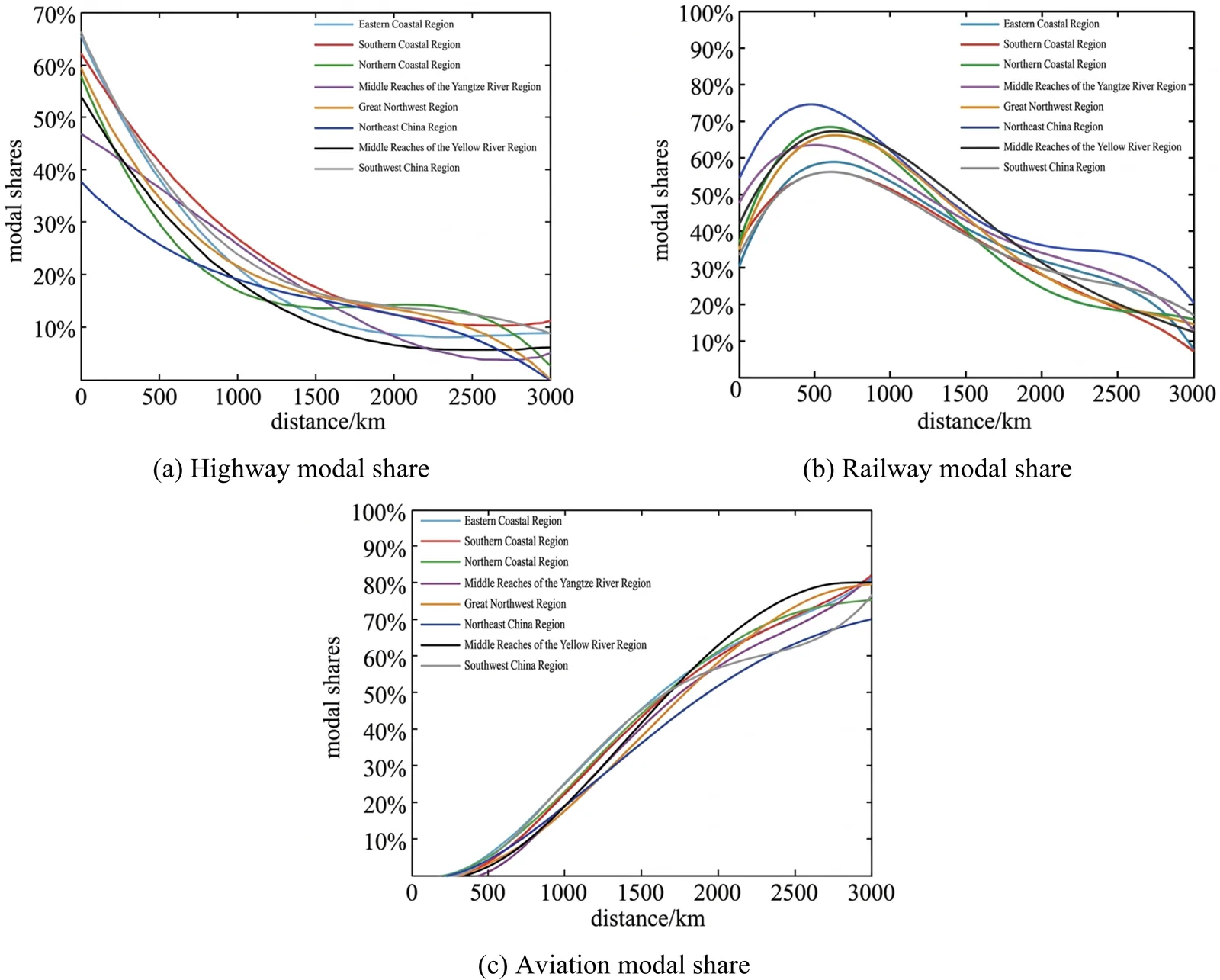
Comparative analysis of modal shares across spatial contexts.

Cities’ differentiated geographical location can influence the travel distances to other cities across the country, which in turn shape intercity passenger modal shares. Based on geographical divisions, all cities in the dataset are categorized into eight regions, namely the Eastern Coastal Region, Southern Coastal Region, Northern Coastal Region, Middle Reaches of the Yangtze River Region, Great Northwest Region, Northeast Region, Middle Reaches of the Yellow River Region and Southwest Region. The modal share models and curves of highway, railway and aviation for cities in each regional category are presented in Table 6 and Fig 7.

**Table 6:** Fitting curves of modal shares across spatial contexts.

Type of City	Traffic mode	The fitted model of intercity travel mode share based on travel distance	R^2^
the eastern coastal region	highway	$f_{\text{1}}\left(x\right)=-3.036\times 10^{-9}x^{\text{3}}+2.474\times 10^{-5}x^{\text{2}}-6.579\times 10^{-2}x+65.44$	0.991
	railway	$f_{\text{2}}\left(x\right)=-8.617\times 10^{-12}x^{\text{4}}+5.894\times 10^{-8}x^{\text{3}}-1.394\times 10^{-4}x^{\text{2}}+0.1132x+29.4$	0.970
	aviation	$\left\{ \begin{array}{c} @l@f_{\text{3}}\left(x\right)=0\;\left(\text{8}\leq x<240\right)\\ f_{\text{3}}\left(x\right)=4.217\times 10^{-12}x^{\text{4}}-2.993\times 10^{-8}x^{\text{3}}+6.617\times 10^{-5}x^{\text{2}}-1.629\times 10^{-2}x+0.5796\;\left(240\leq x\leq 3\text{000}\right) \end{array}\right.$	0.999
the southern coastal region	highway	$f_{\text{1}}\left(x\right)=-1.218\times 10^{-9}x^{\text{3}}+1.395\times 10^{-5}x^{\text{2}}-4.802\times 10^{-2}x+62.29$	0.995
	railway	$f_{\text{2}}\left(x\right)=-4.302\times 10^{-12}x^{\text{4}}+3.27\times 10^{-8}x^{\text{3}}-8.735\times 10^{-5}x^{\text{2}}+7.423\times 10^{-2}x+36.2$	0.999
	aviation	$\left\{ \begin{array}{c} @l@f_{\text{3}}\left(x\right)=0\;\left(\text{8}\leq x<340\right)\\ f_{\text{3}}\left(x\right)=4.186\times 10^{-12}x^{\text{4}}-3.043\times 10^{-8}x^{\text{3}}+7.014\times 10^{-5}x^{\text{2}}-2.252\times 10^{-2}x+0.6782\;\left(340\leq x\leq 3\text{000}\right) \end{array}\right.$	0.999
the northern coastal region	highway	$f_{\text{1}}\left(x\right)=-7.926\times 10^{-9}x^{\text{3}}+4.314\times 10^{-5}x^{\text{2}}-7.669\times 10^{-2}x+58.41$	0.953
	railway	$f_{\text{2}}\left(x\right)=-7.349\times 10^{-12}x^{\text{4}}+5.85\times 10^{-8}x^{\text{3}}-1.54\times 10^{-4}x^{\text{2}}+0.1272x+35.74$	0.987
	aviation	$\left\{ \begin{array}{c} @l@f_{\text{3}}\left(x\right)=0\;\left(\text{8}\leq x<250\right)\\ f_{\text{3}}\left(x\right)=2.192\times 10^{-12}x^{\text{4}}-1.978\times 10^{-8}x^{\text{3}}+5.16\times 10^{-5}x^{\text{2}}-1.095\times 10^{-2}x-0.1624\;\left(250\leq x\leq 3\text{000}\right) \end{array}\right.$	0.999
the middle reaches of the Yangtze River Region	highway	$f_{\text{1}}\left(x\right)=-1.78\times 10^{-9}x^{\text{3}}-3.552\times 10^{-6}x^{\text{2}}-1.941\times 10^{-2}x+46.98$	0.999
	railway	$f_{\text{2}}\left(x\right)=-6.777\times 10^{-12}x^{\text{4}}+4.579\times 10^{-8}x^{\text{3}}-1.05\times 10^{-4}x^{\text{2}}+7.439\times 10^{-2}x+47.19$	0.987
	aviation	$\left\{ \begin{array}{c} @l@f_{\text{3}}\left(x\right)=0\;\left(\text{8}\leq x<490\right)\\ f_{\text{3}}\left(x\right)=5.708\times 10^{-12}x^{\text{4}}-4.047\times 10^{-8}x^{\text{3}}+9.324\times 10^{-5}x^{\text{2}}-4.373\times 10^{-2}x+3.954\;\left(490\leq x\leq 3\text{000}\right) \end{array}\right.$	0.999
the northwestern region	highway	$f_{\text{1}}\left(x\right)=-5.757\times 10^{-9}x^{\text{3}}+3.204\times 10^{-5}x^{\text{2}}-6.406\times 10^{-2}x+59.44$	0.999
	railway	$f_{\text{2}}\left(x\right)=-5.582\times 10^{-12}x^{\text{4}}+4.637\times 10^{-8}x^{\text{3}}-1.294\times 10^{-4}x^{\text{2}}+0.1149x+34.35$	0.980
	aviation	$\left\{ \begin{array}{c} @l@f_{\text{3}}\left(x\right)=0\;\left(\text{8}\leq x<300\right)\\ f_{\text{3}}\left(x\right)=6.013\times 10^{-13}x^{\text{4}}-1.074\times 10^{-8}x^{\text{3}}+3.971\times 10^{-5}x^{\text{2}}-1.238\times 10^{-2}x+0.3631\;\left(300\leq x\leq 3\text{000}\right) \end{array}\right.$	0.999
the northeast Region	highway	$f_{\text{1}}\left(x\right)=-2.995\times 10^{-9}x^{\text{3}}+1.503\times 10^{-5}x^{\text{2}}-3.087\times 10^{-2}x+37.9$	0.983
	railway	$f_{\text{2}}\left(x\right)=-1.007\times 10^{-11}x^{\text{4}}+6.806\times 10^{-8}x^{\text{3}}-1.513\times 10^{-4}x^{\text{2}}+0.1024x+53.07$	0.990
	aviation	$\left\{ \begin{array}{c} @l@f_{\text{3}}\left(x\right)=0\;\left(\text{8}\leq x<270\right)\\ f_{\text{3}}\left(x\right)=1.273\times 10^{-12}x^{\text{4}}-1.223\times 10^{-8}x^{\text{3}}+3.419\times 10^{-5}x^{\text{2}}-3.097\times 10^{-3}x-1.422\;\left(270\leq x\leq 3\text{000}\right) \end{array}\right.$	0.999
the middle reaches of the Yellow River Region	highway	$f_{\text{1}}\left(x\right)=-1.928\times 10^{-9}x^{\text{3}}+1.739\times 10^{-5}x^{\text{2}}-5.087\times 10^{-2}x+54.18$	0.983
	railway	$f_{\text{2}}\left(x\right)=-4.067\times 10^{-12}x^{\text{4}}+3.506\times 10^{-8}x^{\text{3}}-1.028\times 10^{-4}x^{\text{2}}+9.303\times 10^{-2}x+41.07$	0.990
	aviation	$\left\{ \begin{array}{c} @l@f_{\text{3}}\left(x\right)=0\;\left(\text{8}\leq x<360\right)\\ f_{\text{3}}\left(x\right)=1.332\times 10^{-12}x^{\text{4}}-1.705\times 10^{-8}x^{\text{3}}+5.533\times 10^{-5}x^{\text{2}}-2.262\times 10^{-2}x+1.776\;\left(360\leq x\leq 3\text{000}\right) \end{array}\right.$	0.999
the southwest region	highway	$f_{\text{1}}\left(x\right)=-4.644\times 10^{-9}x^{\text{3}}+3.027\times 10^{-5}x^{\text{2}}-6.84\times 10^{-2}x+66.71$	0.999
	railway	$f_{\text{2}}\left(x\right)=-6.083\times 10^{-12}x^{\text{4}}+4.453\times 10^{-8}x^{\text{3}}-1.108\times 10^{-4}x^{\text{2}}+9.058\times 10^{-2}x+32.78$	0.990
	aviation	$\left\{ \begin{array}{c} @l@f_{\text{3}}\left(x\right)=0\;\left(\text{8}\leq x<320\right)\\ f_{\text{3}}\left(x\right)=7.23\times 10^{-12}x^{\text{4}}-4.673\times 10^{-8}x^{\text{3}}+9.37\times 10^{-5}x^{\text{2}}-3.089\times 10^{-2}x+1.742\;\left(320\leq x\leq 3\text{000}\right) \end{array}\right.$	0.999

As depicted in Fig 7(a), highway modal shares exhibit pronounced distance-dependent spatial heterogeneity across Chinese regions. For short-distance trips (<200 km), the Southwest Region, Eastern Coastal Region, and Southern Coastal Region account for relatively higher shares, whereas the Northeast Region records the lowest. In the 200–700 km range, the Southern Coastal Region and Southwest Region maintain relatively high shares, while the Northeast Region remains the least reliant on highway. For medium-to-long distances (700–1500 km), the Southern Coastal Region and Middle Reaches of the Yangtze River Region retain strong highway dominance, whereas the Northern Coastal Region and Middle Reaches of the Yellow River Region show declining shares. Beyond 1500 km, the Northern Coastal Region, Great Northwest Region, and Northeast Region exhibit elevated highway modal shares, reflecting their greater reliance on road transport for long-haul intercity travel.

In Fig 7(b), railway modal shares display distinct regional patterns tied to travel distance. For trips under 1000 km, the Northeast Region, Middle Reaches of the Yellow River Region, and Northern Coastal Region dominate. Notably, the Northeast Region shows a substantially higher share, consistent with its lower reliance on highway travel within this distance range. Between 1000 and 2000 km, the Northern Coastal Region and Great Northwest Region experience a sharp decline in railway modal shares. For distances exceeding 2000 km, the Northeast Region, Middle Reaches of the Yangtze River Region, and Southwest Region maintain relatively high railway shares, while the Northern Coastal Region and Great Northwest Region show reduced reliance on rail.

As shown in Fig 7(c), aviation modal shares are strongly shaped by both travel distance and regional context. For trips below 1,000 km, the Eastern Coastal Region, Northern Coastal Region, and Southern Coastal Region lead in aviation modal shares, whereas the Northeast Region and the Middle Reaches of the Yellow River Region exhibit lower shares. For the 1,000–2,000 km range, aviation modal shares rise rapidly in the Middle Reaches of the Yellow River Region and the Great Northwest Region. Beyond 2,000 km, the Middle Reaches of the Yellow River Region records the highest aviation modal share. Meanwhile, the Great Northwest Region, Southern Coastal Region, and Eastern Coastal Region maintain comparatively strong shares, whereas the Northeast Region and Southwest Region remain lower.

### Comparative analysis in socioeconomic perspective

Cities exhibit distinct socioeconomic attributes, which shape residents’ modal choices and, consequently, determine intercity passenger modal shares. In this section, the dataset is classified into three rounds based on the attributes of departure cities: (i) Economic development level: first-tier, second-tier, third-tier, fourth-tier, and fifth-tier cities; (ii) Administrative rank: provincial-level cities, sub-provincial cities, prefecture-level cities, and county-level cities; (iii) Population size: megacities, supercities, large cities, medium-sized cities, and small cities.

To enable comparative analysis across urban socioeconomic contexts, cities in the dataset are classified according to three dimensions: economic development level, administrative rank, and population size. The classification schemes and corresponding thresholds are detailed in Table 7 below. Importantly, the classification of each city is fixed to the 2018 standard rather than updated annually. This decision is justified by two considerations. First, the city tier and administrative classifications exhibit only marginal year-to-year fluctuations within the 2015–2018 period. Second, fixing the classification to a single reference year eliminates the confounding effect of classification changes over time, thereby isolating the true variation in modal share patterns attributable to travel distance rather than to classification artefacts. This approach aligns with standard practice in longitudinal urban studies, where consistent classification criteria are maintained across study periods to ensure temporal comparability rather than being updated annually [46].

**Table 7:** Socioeconomic classification criteria for cities.

Dimension	Category	Definition/ Threshold
Economic development level	First-tier	Core cities with outstanding economic development
	Second-tier	Major cities with relatively high economic development
	Third-tier	Major cities with moderate economic development
	Fourth-tier	Ordinary cities with developing economies
	Fifth-tier	Less developed cities
Administrative rank	Provincial-level	Municipalities directly under the central government
	Sub-provincial	Provincial capitals and separately planned cities
	Prefecture-level	Standard prefecture-level administrative units
	County-level	County-level administrative units
Population size	Megacities	Urban permanent residents ≥ 10 million
	Supercities	Urban permanent residents 5–10 million
	Large cities	Urban permanent residents 1–5 million
	Medium-sized cities	Urban permanent residents 0.5–1 million
	Small cities	Urban permanent residents < 0.5 million

Note: All classifications are fixed to the 2018 standard to ensure temporal consistency across the 2015–2018 study period. City-tier classifications follow the CBNData China City Business Attractiveness Ranking (2018); administrative ranks follow China’s administrative division system; population size classifications follow the Notice of the State Council on Adjusting the Criteria for City Size Classification (State Council, 2014; Guo Fa No. 51).

#### Influence of economic development level on modal shares.

**Fig 8:**
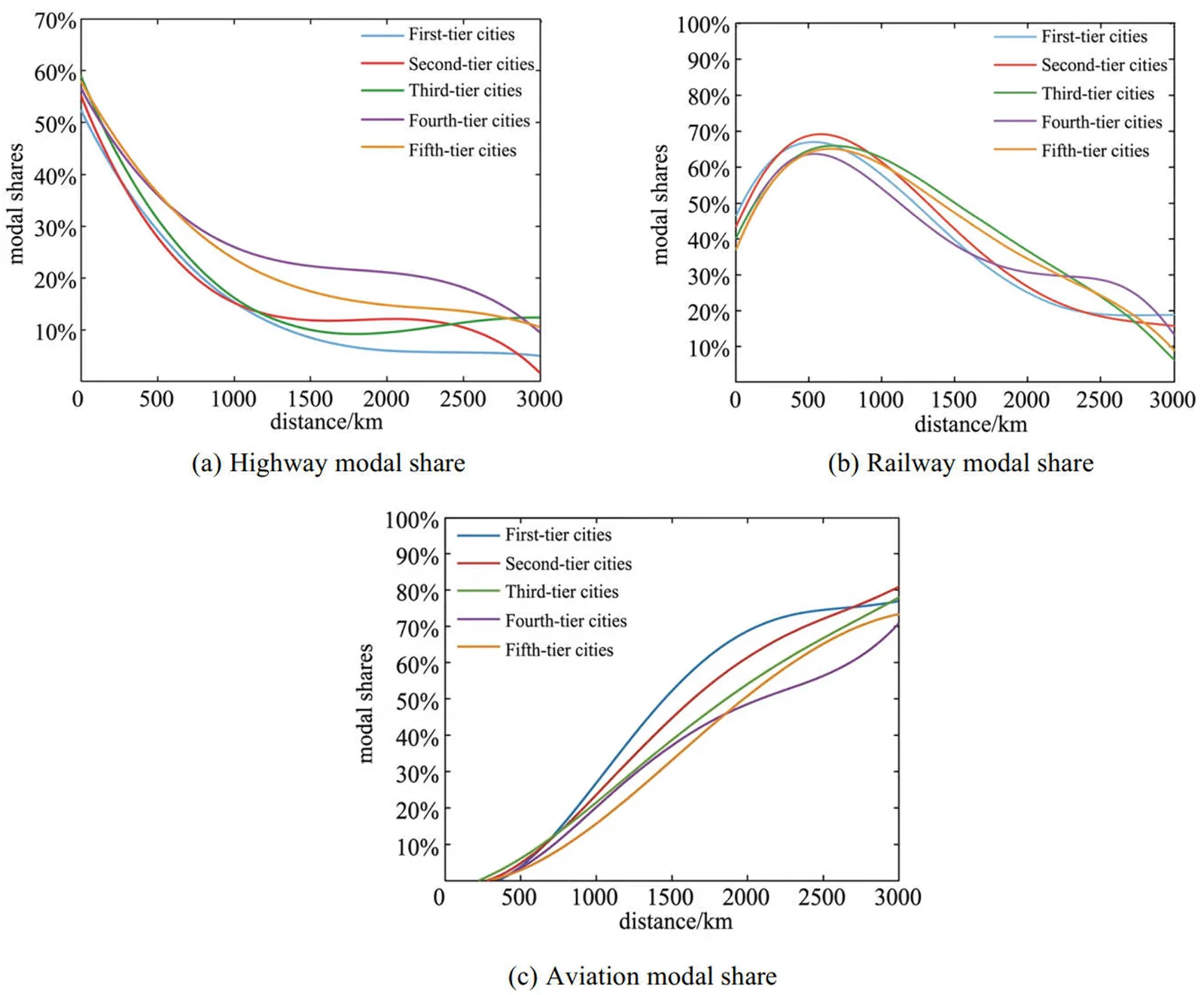
Comparative analysis of modal shares across cities with different economic development levels.

The modal share models and curves of highway, railway, and aviation for cities in each category of economic development level are presented in Table 8 and Fig 8.

**Table 8:** Fitting curves of modal shares across cities with different economic development levels.

Type of City	Traffic mode	The model of intercity travel modal share based on travel distance	R^2^
first-tier cities	highway	$f_{\text{1}}\left(x\right)=-3.128\times 10^{-9}x^{\text{3}}+2.305\times 10^{-5}x^{\text{2}}-5.67\times 10^{-2}x+52.21$	0.997
	railway	$f_{\text{2}}\left(x\right)=-5.102\times 10^{-12}x^{\text{4}}+4.265\times 10^{-8}x^{\text{3}}-1.149\times 10^{-4}x^{\text{2}}+8.963\times 10^{-2}x+45.77$	0.997
	aviation	$\left\{ \begin{array}{c} @l@f_{\text{3}}\left(x\right)=0\;\left(\text{8}\leq x<370\right)\\ f_{\text{3}}\left(x\right)=6.246\times 10^{-12}x^{\text{4}}-4.612\times 10^{-8}x^{\text{3}}+1.039\times 10^{-4}x^{\text{2}}-4.056\times 10^{-2}x+3.031\;\left(370\leq x\leq 3\text{000}\right) \end{array}\right.$	0.998
second-tier cities	highway	$f_{\text{1}}\left(x\right)=-7.287\times 10^{-9}x^{\text{3}}+4.016\times 10^{-5}x^{\text{2}}-7.269\times 10^{-2}x+55.06$	0.968
	railway	$f_{\text{2}}\left(x\right)=-5.458\times 10^{-12}x^{\text{4}}+4.569\times 10^{-8}x^{\text{3}}-1.259\times 10^{-4}x^{\text{2}}+0.1049x+42.25$	0.998
	aviation	$\left\{ \begin{array}{c} @l@f_{\text{3}}\left(x\right)=0\;\left(\text{8}\leq x<290\right)\\ f_{\text{3}}\left(x\right)=3.731\times 10^{-12}x^{\text{4}}-2.803\times 10^{-8}x^{\text{3}}+6.566\times 10^{-5}x^{\text{2}}-1.87\times 10^{-2}x+0.5424\;\left(290\leq x\leq 3\text{000}\right) \end{array}\right.$	0.999
third-tier cities	highway	$f_{\text{1}}\left(x\right)=-4.387\times 10^{-9}x^{\text{3}}+3.102\times 10^{-5}x^{\text{2}}-6.914\times 10^{-2}x+58.77$	0.970
	railway	$f_{\text{2}}\left(x\right)=-4.912\times 10^{-12}x^{\text{4}}+3.687\times 10^{-8}x^{\text{3}}-1.007\times 10^{-4}x^{\text{2}}+9.182\times 10^{-2}x+39.46$	0.987
	aviation	$\left\{ \begin{array}{c} @l@f_{\text{3}}\left(x\right)=0\;\left(\text{8}\leq x<230\right)\\ f_{\text{3}}\left(x\right)=1.481\times 10^{-12}x^{\text{4}}-1.189\times 10^{-8}x^{\text{3}}+2.99\times 10^{-5}x^{\text{2}}+4.002\times 10^{-3}x-2.36\;\left(230\leq x\leq 3\text{000}\right) \end{array}\right.$	0.997
fourth-tier cities	highway	$f_{\text{1}}\left(x\right)=-5.392\times 10^{-9}x^{\text{3}}+2.904\times 10^{-5}x^{\text{2}}-5.433\times 10^{-2}x+56.65$	0.956
	railway	$f_{\text{2}}\left(x\right)=-1.116\times 10^{-11}x^{\text{4}}+7.508\times 10^{-8}x^{\text{3}}-1.684\times 10^{-4}x^{\text{2}}+0.1234x+35.28$	0.984
	aviation	$\left\{ \begin{array}{c} @l@f_{\text{3}}\left(x\right)=0\;\left(\text{8}\leq x<340\right)\\ f_{\text{3}}\left(x\right)=5.514\times 10^{-12}x^{\text{4}}-3.579\times 10^{-8}x^{\text{3}}+7.386\times 10^{-5}x^{\text{2}}-2.52\times 10^{-2}x+1.36\;\left(340\leq x\leq 3\text{000}\right) \end{array}\right.$	0.997
fifth-tier cities	highway	$f_{\text{1}}\left(x\right)=-3.375\times 10^{-9}x^{\text{3}}+2.267\times 10^{-5}x^{\text{2}}-5.335\times 10^{-2}x+57.85$	0.977
	railway	$f_{\text{2}}\left(x\right)=-6.1\times 10^{-12}x^{\text{4}}+4.533\times 10^{-8}x^{\text{3}}-1.189\times 10^{-4}x^{\text{2}}+0.1045x+35.98$	0.996
	aviation	$\left\{ \begin{array}{c} @l@f_{\text{3}}\left(x\right)=0\;\left(\text{8}\leq x<300\right)\\ f_{\text{3}}\left(x\right)=5.234\times 10^{-13}x^{\text{4}}-8.509\times 10^{-9}x^{\text{3}}+3.166\times 10^{-5}x^{\text{2}}-7.991\times 10^{-3}x-0.3945\;\left(300\leq x\leq 3\text{000}\right) \end{array}\right.$	0.999

As illustrated in Fig 8(a), the highway modal share of fourth-tier and fifth-tier cities is generally apparently higher than that of first-tier, second-tier, and third-tier cities; among these, third-tier cities have a higher highway modal share than first-tier and second-tier cities, with first-tier cities recording the lowest. This characteristic is more pronounced for long-distance trips, indicating that the lower the level of economic development of a city, the higher the highway modal share, and vice versa. This is because cities with low economic development are inferior in infrastructure configuration and transport network accessibility compared to those with high economic development. When aviation and railway routes are limited, or even when some cities lack airports or railway stations entirely, trips originating from these cities exhibit a high reliance on highway transport.

According to Fig 8(b), for travel distances less than approximately 700 km, the railway modal share of first- and second-tier cities is obviously higher than that of third-, fourth-, and fifth-tier cities. For distances greater than 1000 km, the railway modal share of third-, fourth-, and fifth-tier cities exceeds that of first- and second-tier cities. In other words, for short-to-medium-distance trips, residents of first- and second-tier cities have a higher proportion of railway travel than those in other cities; for long and ultra-long-distance trips, the railway modal share of third-, fourth-, and fifth-tier cities exceeds that of first- and second-tier cities.

As shown in Fig 8(c), first-tier cities have the highest aviation modal share, followed by second-tier and third-tier cities, indicating that intercity trips originating from cities with higher economic development levels exhibit a greater propensity for aviation. This is consistent with the characteristics of aviation, such as high travel costs and fast speed. For travel distances less than approximately 1800 km, the aviation modal share of fourth-tier cities is higher than that of fifth-tier cities; for distances greater than 1800 km, the proportion of fourth-tier cities choosing aviation is lower than that of fifth-tier cities.

#### Influence of administrative rank on modal shares.

**Fig 9:**
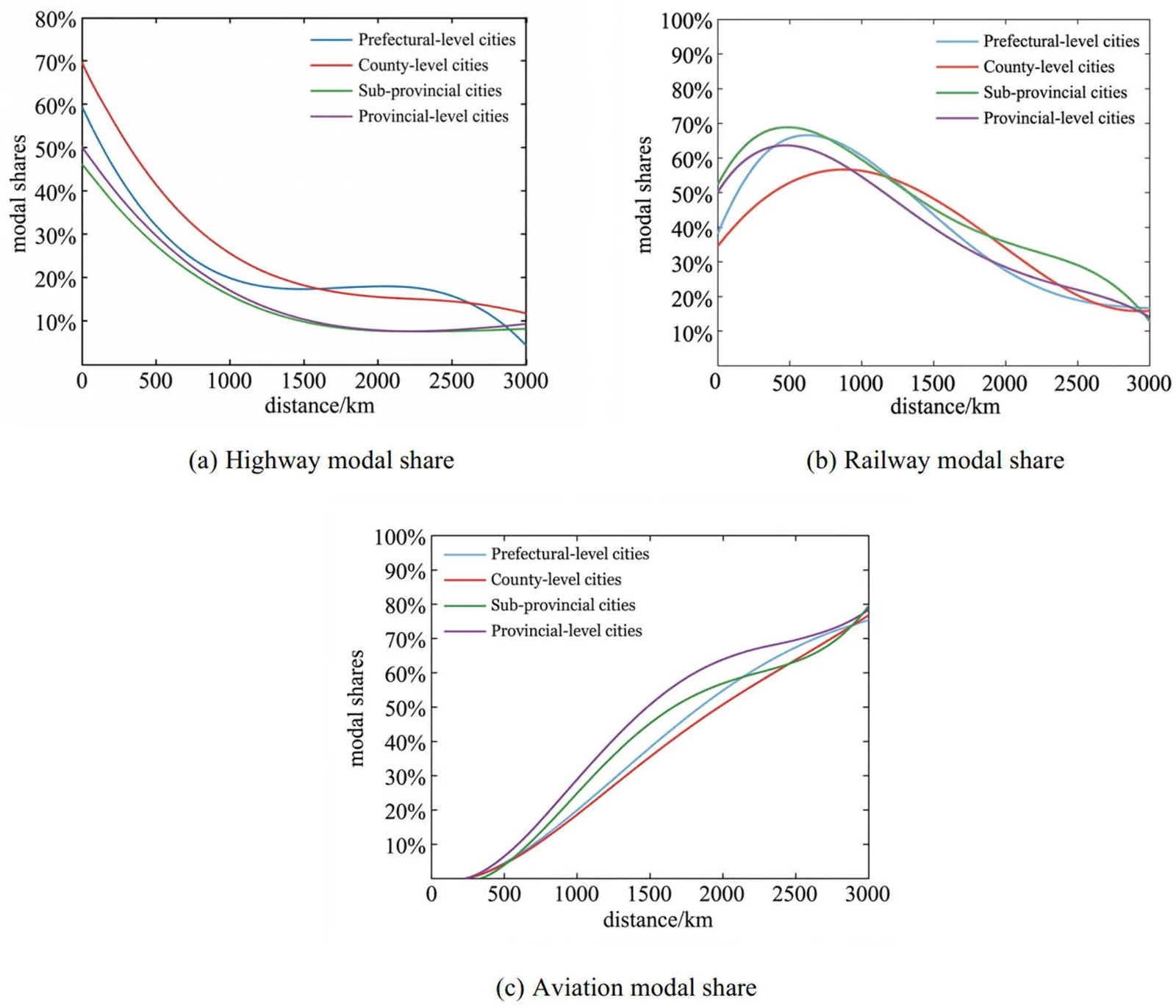
Comparative analysis of modal shares across cities with different administrative rank.

The modal share models and curves of highway, railway and aviation for cities in each category of administrative rank are presented in Table 9 and Fig 9.

**Table 9:** Fitting curves of modal shares across cities with different administrative rank.

Type of City	Traffic mode	The fitted model of intercity travel mode share based on travel distance	R^2^
provincial-level cities	highway	$f_{\text{1}}\left(x\right)=-2.338\times 10^{-9}x^{\text{3}}+1.912\times 10^{-5}x^{\text{2}}-5.016\times 10^{-2}x+50.4$	0.982
	railway	$f_{\text{2}}\left(x\right)=-5.019\times 10^{-12}x^{\text{4}}+3.722\times 10^{-8}x^{\text{3}}-9.195\times 10^{-5}x^{\text{2}}+6.444\times 10^{-2}x+49.92$	0.975
	aviation	$\left\{ \begin{array}{c} @l@f_{\text{3}}\left(x\right)=0\;\left(\text{8}\leq x<260\right)\\ f_{\text{3}}\left(x\right)=6.207\times 10^{-12}x^{\text{4}}-4.198\times 10^{-8}x^{\text{3}}+8.641\times 10^{-5}x^{\text{2}}-2.324\times 10^{-2}x+0.9272\;\left(260\leq x\leq 3\text{000}\right) \end{array}\right.$	0.997
sub-provincial-level cities	highway	$f_{\text{1}}\left(x\right)=-2.259\times 10^{-9}x^{\text{3}}+1.802\times 10^{-5}x^{\text{2}}-4.661\times 10^{-2}x+46.74$	0.996
	railway	$f_{\text{2}}\left(x\right)=-7.482\times 10^{-12}x^{\text{4}}+5.031\times 10^{-8}x^{\text{3}}-1.144\times 10^{-4}x^{\text{2}}+7.933\times 10^{-2}x+51.83$	0.977
	aviation	$\left\{ \begin{array}{c} @l@f_{\text{3}}\left(x\right)=0\;\left(\text{8}\leq x<360\right)\\ f_{\text{3}}\left(x\right)=7.611\times 10^{-12}x^{\text{4}}-4.878\times 10^{-8}x^{\text{3}}+9.762\times 10^{-5}x^{\text{2}}-3.343\times 10^{-2}x+1.43\;\left(360\leq x\leq 3\text{000}\right) \end{array}\right.$	0.997
prefecture-level cities	highway	$f_{\text{1}}\left(x\right)=-8.263\times 10^{-9}x^{\text{3}}+4.389\times 10^{-5}x^{\text{2}}-7.585\times 10^{-2}x+60.07$	0.914
	railway	$f_{\text{2}}\left(x\right)=-4.847\times 10^{-12}x^{\text{4}}+4.215\times 10^{-8}x^{\text{3}}-1.208\times 10^{-4}x^{\text{2}}+0.107x+37.19$	0.976
	aviation	$\left\{ \begin{array}{c} @l@f_{\text{3}}\left(x\right)=0\;\left(\text{8}\leq x<280\right)\\ f_{\text{3}}\left(x\right)=1.309\times 10^{-12}x^{\text{4}}-1.264\times 10^{-8}x^{\text{3}}+3.585\times 10^{-5}x^{\text{2}}-3.586\times 10^{-3}x-1.53\;\left(280\leq x\leq 3\text{000}\right) \end{array}\right.$	0.999
county-level cities	highway	$f_{\text{1}}\left(x\right)=-4.664\times 10^{-9}x^{\text{3}}+3.104\times 10^{-5}x^{\text{2}}-7.061\times 10^{-2}x+69.9$	0.977
	railway	$f_{\text{2}}\left(x\right)=1.904\times 10^{-12}x^{\text{4}}+1.484\times 10^{-9}x^{\text{3}}-3.418\times 10^{-5}x^{\text{2}}+5.359\times 10^{-2}x+34.15$	0.995
	aviation	$\left\{ \begin{array}{c} @l@f_{\text{3}}\left(x\right)=0\;\left(\text{8}\leq x<250\right)\\ f_{\text{3}}\left(x\right)=2.205\times 10^{-12}x^{\text{4}}-1.652\times 10^{-8}x^{\text{3}}+4.098\times 10^{-5}x^{\text{2}}-8.12\times 10^{-3}x-0.3274\;\left(250\leq x\leq 3\text{000}\right) \end{array}\right.$	0.99

As illustrated in Fig 9(a), provincial-level and sub-provincial-level cities register the lowest highway modal share, followed by prefecture-level and county-level cities. Specifically, the highway modal share curves of sub-provincial-level and provincial-level cities exhibit a high degree of similarity; the key disparity lies in that for travel distances less than 1500 km, provincial-level cities have a higher highway modal share than sub-provincial-level ones, with the gap becoming more pronounced as travel distances shorten. By contrast, the proportion of highway travel in county-level and prefecture-level cities is observably higher than that in provincial-level and sub-provincial-level cities. Overall, county-level cities have the highest highway modal share: particularly for travel distances under 1500 km, residents of county-level cities opt for highway transport at a far higher rate than those in the other three categories. For distances exceeding 1500 km, the average highway modal share of county-level and prefecture-level cities converges to a similar level.

As shown in Fig 9(b), sub-provincial-level cities have a higher railway modal share than the other city categories. In detail, for travel distances less than approximately 450 km, sub-provincial-level cities rank first in railway modal share, followed by provincial-level, prefecture-level and county-level cities in that order. According to Fig 9(c), provincial-level cities boast the highest aviation modal share, with sub-provincial-level, prefecture-level and county-level cities following successively.

#### Influence of population size on modal shares.

**Fig 10:**
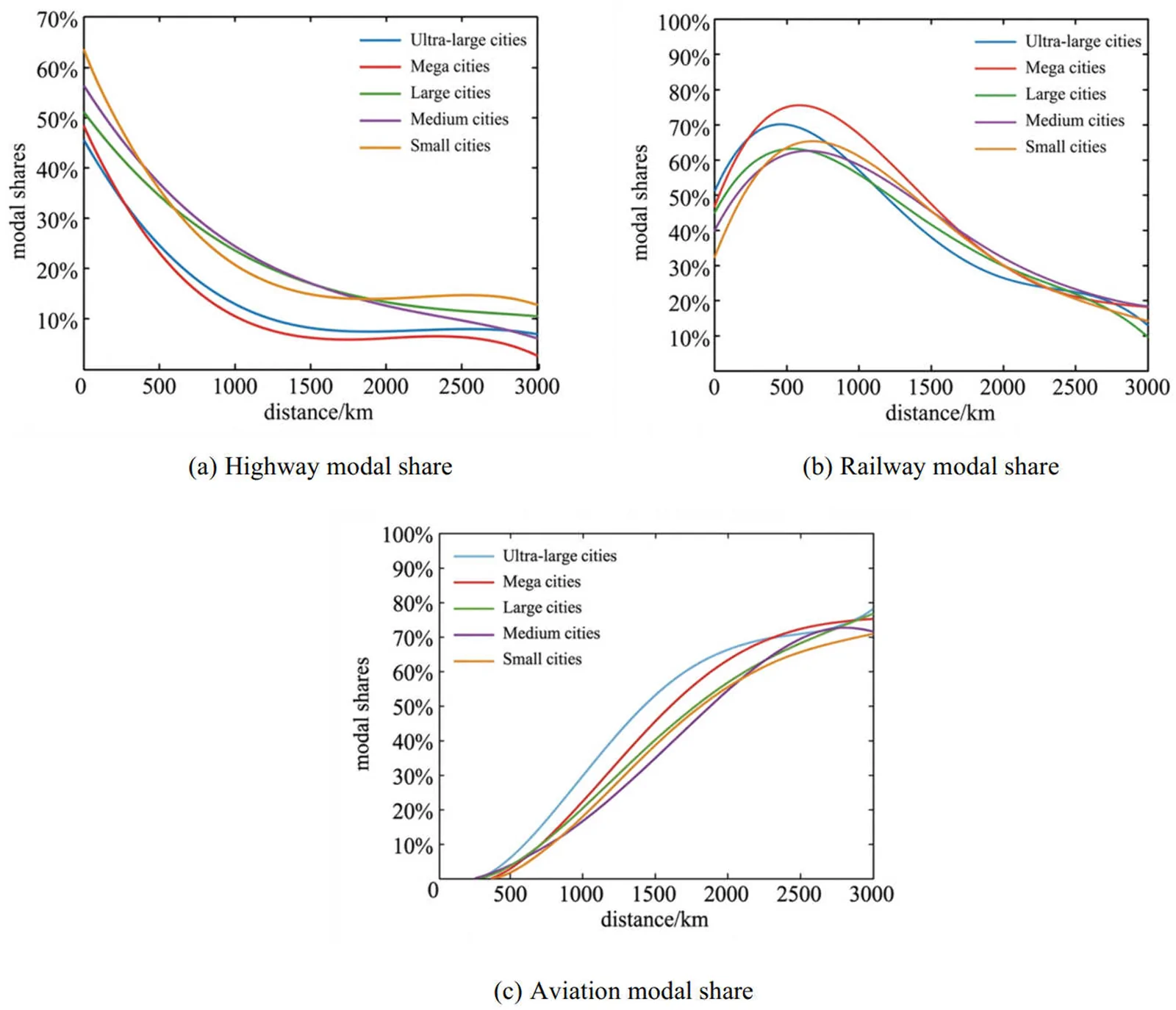
Comparative analysis of modal shares across cities with different population size.

The modal share models and curves of highway, railway and aviation for cities in each category of population size are presented in Table 10 and Fig 10.

**Table 10:** Fitting curves of modal shares across cities with different population size.

Type of City	Traffic mode	The fitted model of intercity travel mode share based on travel distance	R^2^
megacities	highway	$f_{\text{1}}\left(x\right)=-5.442\times 10^{-9}x^{\text{3}}+3.314\times 10^{-5}x^{\text{2}}-6.576\times 10^{-2}x+48.68$	0.985
	railway	$f_{\text{2}}\left(x\right)=-5.875\times 10^{-12}x^{\text{4}}+4.921\times 10^{-8}x^{\text{3}}-1.357\times 10^{-4}x^{\text{2}}+0.1134x+46.4$	0.984
	aviation	$\left\{ \begin{array}{c} @l@f_{\text{3}}\left(x\right)=0\;\left(\text{8}\leq x<370\right)\\ f_{\text{3}}\left(x\right)=4.027\times 10^{-12}x^{\text{4}}-3.258\times 10^{-8}x^{\text{3}}+8.026\times 10^{-5}x^{\text{2}}-3.208\times 10^{-2}x+2.447\;\left(370\leq x\leq 3\text{000}\right) \end{array}\right.$	0.994
super cities	highway	$f_{\text{1}}\left(x\right)=-3.673\times 10^{-9}x^{\text{3}}+2.468\times 10^{-5}x^{\text{2}}-5.411\times 10^{-2}x+46.2$	0.977
	railway	$f_{\text{2}}\left(x\right)=-8.653\times 10^{-12}x^{\text{4}}+6.092\times 10^{-8}x^{\text{3}}-1.406\times 10^{-4}x^{\text{2}}+9.449\times 10^{-2}x+50.97$	0.996
	aviation	$\left\{ \begin{array}{c} @l@f_{\text{3}}\left(x\right)=0\;\left(\text{8}\leq x<300\right)\\ f_{\text{3}}\left(x\right)=6.886\times 10^{-12}x^{\text{4}}-4.651\times 10^{-8}x^{\text{3}}+9.508\times 10^{-5}x^{\text{2}}-2.629\times 10^{-2}x+0.5921\;\left(300\leq x\leq 3\text{000}\right) \end{array}\right.$	0.998
large cities	highway	$f_{\text{1}}\left(x\right)=-1.609\times 10^{-9}x^{\text{3}}+1.34\times 10^{-5}x^{\text{2}}-3.934\times 10^{-2}x+51.33$	0.987
	railway	$f_{\text{2}}\left(x\right)=-5.683\times 10^{-12}x^{\text{4}}+4.119\times 10^{-8}x^{\text{3}}-1.023\times 10^{-4}x^{\text{2}}+7.793\times 10^{-2}x+44.84$	0.983
	aviation	$\left\{ \begin{array}{c} @l@f_{\text{3}}\left(x\right)=0\;\left(\text{8}\leq x<300\right)\\ f_{\text{3}}\left(x\right)=2.82\times 10^{-12}x^{\text{4}}-2.24\times 10^{-8}x^{\text{3}}+5.57\times 10^{-5}x^{\text{2}}-1.625\times 10^{-2}x+0.3416\;\left(300\leq x\leq 3\text{000}\right) \end{array}\right.$	0.999
medium-sized cities	highway	$f_{\text{1}}\left(x\right)=-2.516\times 10^{-9}x^{\text{3}}+1.763\times 10^{-5}x^{\text{2}}-4.711\times 10^{-2}x+56.57$	0.990
	railway	$f_{\text{2}}\left(x\right)=-3.457\times 10^{-12}x^{\text{4}}+3.03\times 10^{-8}x^{\text{3}}-8.934\times 10^{-5}x^{\text{2}}+8.142\times 10^{-2}x+39.68$	0.980
	aviation	$\left\{ \begin{array}{c} @l@f_{\text{3}}\left(x\right)=0\;\left(\text{8}\leq x<260\right)\\ f_{\text{3}}\left(x\right)=-1.37\times 10^{-12}x^{\text{4}}+1.469\times 10^{-9}x^{\text{3}}+1.492\times 10^{-5}x^{\text{2}}+3.349\times 10^{-3}x-1.891\;\left(260\leq x\leq 3\text{000}\right) \end{array}\right.$	0.999
small cities	highway	$f_{\text{1}}\left(x\right)=-5.123\times 10^{-9}x^{\text{3}}+3.343\times 10^{-5}x^{\text{2}}-7.112\times 10^{-2}x+63.69$	0.978
	railway	$f_{\text{2}}\left(x\right)=-5.216\times 10^{-12}x^{\text{4}}+4.379\times 10^{-8}x^{\text{3}}-1.251\times 10^{-4}x^{\text{2}}+0.1162x+31.47$	0.995
	aviation	$\left\{ \begin{array}{c} @l@f_{\text{3}}\left(x\right)=0\;\left(\text{8}\leq x<400\right)\\ f_{\text{3}}\left(x\right)=3.326\times 10^{-12}x^{\text{4}}-2.734\times 10^{-8}x^{\text{3}}+6.982\times 10^{-5}x^{\text{2}}-3.0525\times 10^{-2}x+2.593\;\left(400\leq x\leq 3\text{000}\right) \end{array}\right.$	0.999

As illustrated in Fig 10(a), small, medium and large cities register a substantially higher proportion of highway travel for intercity trips compared to megacities and super-megacities. For travel distances below approximately 500 km, small cities exhibit a markedly higher highway modal share than cities in the other four categories. For distances exceeding around 500 km, the highway modal share of medium and small cities experiences a relatively rapid decline, whereas that of small cities decreases at a moderate pace—particularly for the distance range of 1500–3000 km, where the highway modal share sees negligible fluctuations.

As shown in Fig 10(b), for travel distances below about 800 km, super-megacities and megacities exhibit apparently higher railway modal shares than large, medium, and small cities. Beyond this range, the railway modal shares of super-megacities gradually decline and fall below those of other city categories. Notably, it is not until travel distances reach approximately 1,700 km that the railway modal share of megacities converges with those of other city types. According to Fig 10(c), Residents of super-megacities exhibit a pronounced preference for aviation compared with residents in other city categories. This preference is followed by megacities and large cities, whereas residents in medium and small cities display comparable average aviation modal shares.

## Discussion on multiscale comparisons

Intercity passenger modal shares for highway, railway, and aviation exhibit consistent distance- dependent patterns, with clear threshold differences across modes. These dynamics are driven by shared underlying mechanisms, yet differ in their sensitivity to distance, and show substantial heterogeneity shaped by temporal contexts, geographical location, and urban socioeconomic attributes. Collectively, these findings characterize the competitive and complementary relationships among highway, railway, and aviation transport.

Temporally, holiday periods reshape modal share thresholds while preserving distance sensitivity. For highway transport, modal share is observably higher during these periods than in regular periods for short-to-medium trips (≤450 km for weekends, ≤ 1100 km for Golden Week, ≤ 900 km for SFTR) but declines for long-distance trips. This duality stems from three core drivers: trips during holidays are predominantly non-business-oriented, with travelers prioritizing the “door-to-door” convenience of highway transport for short distances; nationwide toll-free highway policies during Golden Week and SFTR substantially reduce travel costs, incentivizing highway use; highways are less competitive than high-speed rail and aviation for long trips due to lower speed and longer travel time, compounded by business travelers—more prevalent in regular periods—exhibiting a stronger preference for rail/aviation. In contrast, railway modal share follows an opposing trend: it is lower during holidays for short-to-medium distances (≤600 km for Golden Week, ≤ 900 km for weekends) due to highway competition and ticket shortages, with SFTR causing steeper drops beyond 600 km. The influence of holidays on aviation modal shares is primarily affected in long-distance trips. During Golden Week, aviation modal share is lower for trips of ≤1500 km but higher for trips ranging from 1500 to 2750 km. This variation is driven by travelers’ demand for time efficiency, as they aim to maximize their limited holiday opportunities for leisure, tourism, or family visits, making aviation—the fastest long-distance transport mode—a preferred choice for journeys within the 1500–2750 km range. In contrast, the SFTR consistently boosts aviation modal share for trips of >1500 km. This is attributed to the surge in intercity travel demand during the SFTR, coupled with aviation’s inherent advantage, which enables travelers to shorten their journey time and better meet the entertainment demand during holiday periods.

Seasonal variations exert a more pronounced impact than the weekday-weekend distinction, with spring/summer and autumn/winter forming clustered trends. For highways, autumn and winter exhibit higher modal shares than spring and summer for trips up to 1500 km. This pattern may be partly attributable to colder weather conditions, which could encourage travelers to opt for self-driving to avoid the inconvenience of public transit transfers, although this interpretation remains speculative without direct behavioural data. In contrast, spring and summer present higher highway modal shares for trips exceeding 1500 km, as favorable weather conditions enhance overall road safety and driving reliability. Winter registers the highest highway modal share for trips up to 650 km, which is largely attributed to the fact that the SFTR typically falls within winter, generating intensive short-to-medium-distance highway travel demand. Railway exhibits distance-dependent seasonal dominance: autumn leads for 1200–2600 km, winter for trips >2600 km, and spring/summer for trips ≤1000 km. A strong negative correlation between railway and highway shares is observed for trips ≤1200 km across all seasons. Aviation’s seasonal effects are concentrated in long-distance trips exceeding 1500 km, within which winter yields an apparently higher aviation modal share. One possible interpretation is that adverse winter road conditions may reduce the attractiveness of highway travel for long-distance journeys, potentially leading some travelers to switch to aviation; however, this causal link cannot be directly established from the present data. Spring, summer, and autumn exhibit similar patterns in aviation modal share, with autumn recording the highest share for trips between 1,700 and 3,000 km. For trips of 1,500 km or less, no significant seasonal variation in aviation modal share is observed.

Spatially, geographical location amplifies the heterogeneity in intercity passenger modal shares, with regional modal preferences varying with travel distance and being inherently shaped by terrain, climatic features and transport infrastructure endowment. For highway transport, the Southwest Region exhibits the highest highway modal share for short-distance trips (≤200 km). While this pattern may be partly associated with the region’s complex terrain, it is also important to note that the use of great-circle distance as a unified metric may introduce non-negligible measurement errors for highway travel in such mountainous areas, potentially overestimating the distance decay effect for road transport relative to other modes. The Southern Coastal, Southwest, Eastern Coastal, Middle Reaches of the Yangtze River and Great Northwest Regions maintain robust highway shares for 200–1500 km, owing to either well-developed road systems or limited alternative transport capacity for medium-distance travel. For long trips (>1500 km), the Northern Coastal, Great Northwest and Northeast Regions rely more on highways. This observation is suggestive of potentially limited long-haul aviation and rail accessibility in these regions, though this interpretation should be treated as a plausible hypothesis rather than a confirmed causal relationship.

Railway modal shares present a regional pattern complementary to highway preferences, with the Northeast Region exhibiting a distinctive and persistent railway dominance across all distance bands. This region records the highest railway share for trips ≤1000 km, which aligns with its lowest highway reliance in this range, and retains strong railway competitiveness for trips >2000 km. This pattern is consistent with the region’s well-established industrial railway network and harsh winter climates that tend to reduce highway usability. In contrast, the Northern Coastal, Great Northwest and Middle Reaches of the Yellow River Regions see a sharp decline in railway shares for 1000–2000 km, as travelers shift to more competitive aviation or highway options for medium-long haul travel.

Aviation modal shares are markedly higher in the Eastern, Northern and Southern Coastal Regions for trips ≤1000 km, which is consistent with the advanced aviation infrastructure and the concentration of short-haul business-related trips originating from these regions. The Middle Reaches of the Yellow River and Great Northwest Regions witness rapid aviation share growth for 1000–2000 km, as improved aviation capacity mitigates the inefficiency of highway and railway for medium-long trips. The Middle Reaches of the Yellow River Region further emerges as the aviation leader for trips >2000 km due to its central geographic location as a national aviation hub. The Northeast Region lags notably in both highway share for trips ≤1000 km and aviation share for trips >1000 km relative to other regions, a unique spatial feature rooted in its rail-centric infrastructure layout, climatic constraints on road travel and underdeveloped aviation networks.

From the socioeconomic perspective, modal shares are stratified by urban attributes—economic development level, administrative rank, and population size—exerting a hierarchical influence: higher economic level, administrative rank, and population size correlate with higher aviation share and lower highway share, and vice versa. Fourth/fifth-tier, county-level, and small cities rely most on highways, even for long-distance trips, due to limited railway and aviation infrastructure. Railway modal share exhibits a distinct distance-dependent reversal. For trips of 700–800 km or shorter, first- and second-tier cities and megacities demonstrate a higher railway share, owing to their dense and well‑developed short‑haul rail networks that ensure high accessibility and service frequency. By contrast, third-, fourth-, and fifth-tier cities dominate in railway share for trips exceeding 1000 km, primarily because these cities are generally equipped with limited aviation infrastructure and scarce flight options. Aviation follows a clear hierarchy (first-tier > second-tier > third-tier > fourth-tier > fifth-tier) with a minor reversal between fourth and fifth tiers for trips >1800 km. This hierarchical pattern is suggestive of a potential association with economic capacity and the high-cost, high-speed attributes of air travel. Administrative rank reinforces these patterns: provincial-level cities boast the highest aviation share, while county-level cities lead in highway use for trips ≤1500 km, reflecting gaps in infrastructure investment.

Overall, distance dependence serves as a foundational pattern governing intercity transport modal shares, while spatiotemporal contexts and urban socioeconomic attributes introduce heterogeneity. These findings offer a nuanced understanding of intercity mode dynamics, thereby informing targeted optimization of intercity transport systems and balanced regional development.

## Conclusions and future outlooks

This study conducts a comprehensive and in-depth analysis of intercity passenger transport modal shares, leveraging large-scale Tencent LBS big data covering 362 cities in China from 2015 to 2018. Compared with traditional OD surveys and questionnaire-based studies, this dataset offers broader coverage, higher spatiotemporal precision, and more representative insights into real-world travel behavior. Taking travel distance as the core explanatory variable, the study constructs polynomial fitting models for modal share curves of highway, railway, and aviation, and systematically examines the heterogeneous effects across temporal dimensions (seasons, weekdays/weekends, Golden Week, SFTR), spatial dimensions (geographical location), and socioeconomic dimensions (economic development level, administrative rank, population size). This research framework effectively reveals the nonlinear relationship between travel distance and modal share, as well as multiscale heterogeneity of transport mode choices, providing a robust quantitative basis for intercity transport planning. Main findings of this study are summarized below:

Temporal contexts exert differentiated effects on intercity passenger modal shares, with seasonal variation and the Spring Festival Travel Rush (SFTR) showing stronger influences than weekday–weekend differences. Highway modal shares are higher on weekends for trips ≤450 km, during Golden Week for trips ≤1100 km, and during the SFTR for trips ≤900 km. Correspondingly, railway modal shares decrease within these distance ranges, while railway shares during the SFTR are substantially lower for trips exceeding 600 km. Aviation modal shares are higher during Golden Week for trips between 1500 and 2750 km and during the SFTR for trips longer than 1500 km. Seasonal patterns show that spring and summer exhibit similar modal share curves, as do autumn and winter. In addition, highway modal shares are higher in autumn and winter for trips ≤1500 km, whereas aviation modal shares are higher in winter for trips >1500 km. Spatially, the Northeast Region consistently exhibits the highest railway modal shares for trips ≤1000 km, coastal regions record relatively higher aviation modal shares for trips ≤1000 km, and the Southwest Region shows the highest highway modal shares for trips shorter than 200 km. These results demonstrate substantial temporal and spatial heterogeneity in intercity passenger modal shares.

Socioeconomic heterogeneity is also associated with distinct intercity passenger modal share patterns. Higher urban economic development levels, administrative ranks, and population sizes are associated with higher aviation modal shares and lower highway modal shares. Fourth- and fifth-tier cities, county-level cities, and small- and medium-sized cities record the highest highway modal shares, particularly for trips within 500 km. In contrast, first-tier cities, provincial-level cities, and megacities exhibit relatively higher railway modal shares for short- to medium-distance trips, whereas third- to fifth-tier cities, county-level cities, and small- and medium-sized cities show higher railway modal shares for trips exceeding 1000 km. Aviation modal shares increase systematically with urban development level, with first-tier cities recording the highest aviation shares for trips beyond 1800 km. Overall, urban socioeconomic characteristics exhibit a notable influencing effect on the distance-modal share relationship.

Travel distance is the dominant factor governing intercity passenger modal shares and determines the competitive relationships among transport modes. For short- to medium-distance trips, modal competition is primarily between highway and railway, whereas for long- and ultra-long-distance trips it shifts to railway and aviation. This distance-dependent pattern remains robust across temporal, spatial, and socioeconomic contexts, although the distance thresholds and modal share levels vary substantially among different contexts. Guo et al. [47] found the consistent distance–modal share patterns with our findings based on 27,191 survey responses from a single city, i.e., highway dominance for short trips and aviation preference for long trips. The convergence between our LBS-derived estimates and these independent findings substantiates the external validity and representativeness of our dataset for characterising intercity modal share patterns.

Several limitations warrant further investigation. First, the polynomial fitting approach treats each modal share independently without explicitly accounting for the compositional constraint that the three shares sum to 100%. While this approach is appropriate for the descriptive objective of this study and achieves high goodness-of-fit, future research could adopt compositional modeling frameworks that jointly estimate modal shares while preserving the sum-to-one constraint. Second, this study considers only three representative transport modes—highway, railway, and aviation—and excludes other travel modes, such as water transport, which may be important in specific regions. Third, although travel distance is the primary explanatory variable, individual-level characteristics are not considered. Incorporating traveler heterogeneity would provide a more comprehensive understanding of intercity mode choice. Finally, the use of great-circle distance, while ensuring cross-modal comparability, may introduce measurement errors because actual highway and railway travel distances often deviate from straight-line distances, particularly in mountainous regions. Future studies could incorporate mode-specific network distances to further improve the robustness of regional comparisons.
